# Exploring the influence of mesoporosity in hard carbon-templated hierarchical SAPO-5 for ethanol dehydration†

**DOI:** 10.1039/d4lf00230j

**Published:** 2024-08-08

**Authors:** Matthew E. Potter, Evangeline B. McShane, Nienke L. Visser, Johannes D. Meeldijk, Lisa J. Allen, Stephen M. King, Marina Carravetta, Petra E. de Jongh, Bart D. Vandegehuchte, Robert Raja

**Affiliations:** a UK CatalysisHub, Research Complex at Harwell, Rutherford Appleton Laboratory Didcot OX11 0FA UK mep61@bath.ac.uk; b Chemistry Department, University College London 20 Gordon Street London WC1H 0AJ UK; c School of Chemistry, University of Southampton Highfield Campus Southampton SO17 1BJ UK; d Materials Chemistry and Catalysis, Debye Institute for Nanomaterials Science, Utrecht University Universiteitsweg 99 3584 CG Utrecht The Netherlands; e ISIS Neutron and Muon Source, STFC Rutherford Appleton Laboratory Harwell Campus Didcot OX11 0QX UK; f TotalEnergies, OneTech Belgium Zone Industrielle Feluy C B-7181 Seneffe Belgium

## Abstract

Microporous solid acid catalysts offer a vast amount of control over chemical processes. However, their coveted smaller pores also have several drawbacks, including a limited substrate scope, faster deactivation, and pore blockage. As such, there are significant advantages to introducing mesopores alongside the microporous framework, to create hierarchically porous frameworks. This work explores the influence of adapting our microporous synthetic procedure for silicoaluminophosphate (SAPO-5) to include different shaped carbon nanotemplates. The differing size of the mesopores formed is explored using nitrogen physisorption, transmission electron microscopy and small angle neutron scattering. In this work, we uniquely use small angle neutron scattering for probing hierarchical silicoaluminophosphates synthesised with hard templating methods. Here small angle neutron scattering was able to probe the shape and size of the mesopores and link their accessibility to their catalytic performance.

## Abstract 2

Carbon nanotemplates are used to create hierarchical silicoaluminophosphate materials, with superior catalytic performance to microporous materials. Their hierarchical nature is probed with in-depth microscopy and small-angle neutron scattering.

## Graphical abstract

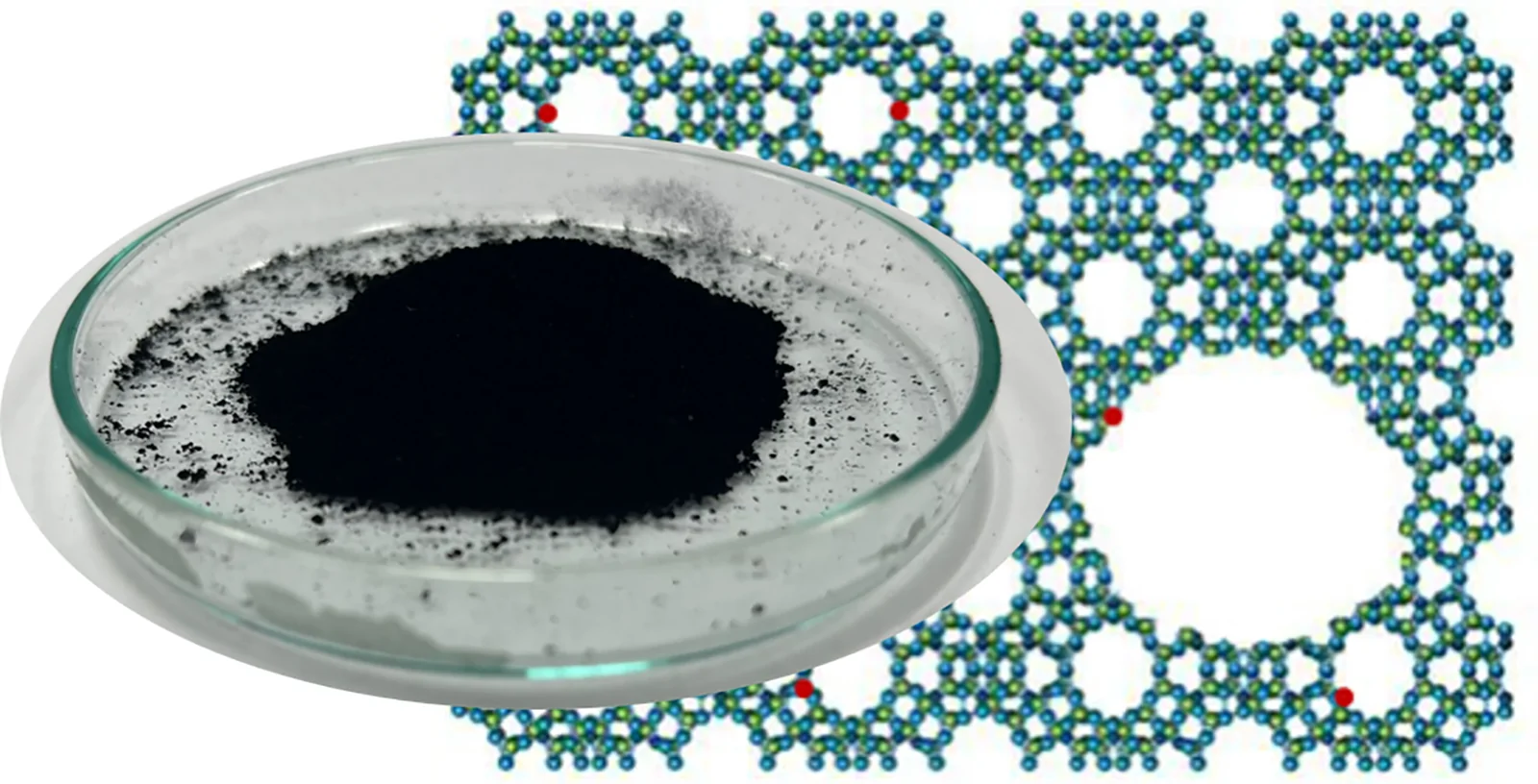

## Introduction

Solid acid catalysts are a vital component for a wide range of bulk chemical processes, including hydrocarbon cracking,^1^ dewaxing,^2^ and olefin production,^3^ the products of which continue to play a major role in modern life. Further, these three processes all use porous solid-acid catalysts, where the pore sizes are less than 20 Å, specifically making them microporous catalysts. Microporous catalysts, also known as “molecular sieves”, offer a huge amount of control over the progression of a reaction, due to the large number of catalytic active sites within these pores.^4^ The pores are often similar in size to small molecules, meaning that large or “bulky” molecules, often cannot diffuse into the pores, preventing access to the catalytic active sites inside and stopping them from reacting.^5,6^ Similarly, the confined environment in the pores is known to influence the precise reaction pathway followed, as it can increase the energy barrier of larger transition states, tailoring the formation of specific products.^7^ A further benefit is that if large molecules form within the pore, microporous materials have the ability to “trap” these molecules within a pore, close to an active site. This then forces the molecule to react further, into a species that is sufficiently small to diffuse out of the pores.^8^ Zeolites (aluminosilicates) are among the most common class of microporous catalysts and are widely used in industry.^9^ These materials are made from tetrahedral Si or Al species, that combine to make a crystalline framework, where the precise topology is dictated by the number of unique tetrahedral units, their bond angles, and lengths.^10,11^

Aluminophosphates (AlPOs) are similar to zeolites, and often form identical frameworks. Unlike zeolites AlPOs are constructed from alternating AlO_4_ and PO_4_ tetrahedra, joined through Al–O–P bonds.^12^ AlPOs themselves are comparatively inactive species, even though the surface is decorated with defect Al–OH and P–OH species, which are incredibly weak acid sites. By substituting heteroatoms into the framework, in the place of Al or P atoms, a variety of active sites can be created.^13–15^ While many different heteroatoms have been incorporated into AlPOs, the most common dopant is Si, forming a silicon-doped aluminophosphate (SAPO), leading to the creation of a Brønsted acid site (BAS).^16^ Substituting a framework P^5+^ site for a Si^4+^ dopant, creates a charge imbalance, which is countered by a proton binding to an oxygen, adjacent to the Si^4+^ dopant, forming a BAS.^17^ Many studies have shown that while the choice of framework influences the reaction pathway (as previously discussed), it also influences the acidic properties of the BAS formed in SAPOs,^13,16,18^ making it important to carefully control the framework topology.

Whilst microporous materials offer significant advantages, their smaller pores can bring several disadvantages. Their molecular sieve properties are highly beneficial for smaller molecules, but this greatly limits the substrate scope for these catalysts. This is particularly unfortunate considering the growing interest in lignin, sugars and other biomass-based feedstocks.^19,20^ Another issue is pore diffusion. Several studies have shown that the pores are often not uniformly filled across the whole particle.^21–23^ Instead, only pores close to the surfaces are filled, lowering the efficiency of the catalyst. Finally smaller pores are more susceptible to blockage, which limits access to the active sites, reducing the catalysts activity, leading to deactivation.^24–26^ This is particularly challenging in processes which go *via* bulkier intermediates, such as the methanol-to-olefin (MTO) process, where bulky aromatics are a vital component of the reaction pathway.^24–26^ As such there is significant interest in introducing larger pores into SAPO frameworks, to improve pore diffusion and negate these issues.

Adding either mesopores or macropores (pore sizes of 20 to 500 Å, or >500 Å respectively) into a microporous system leads to the formation of hierarchically porous (HP) systems. HP-zeolites are regularly formed by “etching” under acidic or basic conditions.^27–29^ This approach has previously been followed using electron tomography^30^ to visualise the mesopores within hierarchical zeolites, and their linkage to the systems micropores, by combining multiple images of the mesopore network. Despite focussing on a small volume of sample, it is possible to both visualise and quantify the degree of accessibility of different mesopores, and relate this to catalytic activity, something previously analysed in zeolite Y for hydrocracking.^31,32^ AlPOs and SAPOs are not as resilient to changes in pH as zeolites,^33^ so often use a “dual template” approach to form HP systems. Here the framework forms around a mesopore template, as the microporous framework forms around the amine or ammonium micropore template.^34,35^ Mesopore templates are either “hard” or “soft”. A hard template is rigid and pre-formed, examples of which are polystyrene spheres, biological materials, and carbon particles.^34,35^ In contrast soft templates self-assemble into a particular shape during the synthesis.

Interest in hard-templated SAPOs has mainly focussed on incorporating mesopores into SAPO-34 (a chabazite system; framework code CHA), to improve its lifetime and performance for the commercial MTO process.^36–41^ Typically the inclusion of mesopores was found to lower the total surface area of the system, whilst increasing the total pore volume, due to a significant increase in the mesopore volume.^40–42^ The inclusion of mesopores rarely increases the number of BAS, though shows improved catalytic lifetime over conventional SAPO-34 species.^40–42^ Investigations have also considered multi-walled carbon nanotubes (MWCNT),^41,43^ pearls,^44^ carbon nanofibers^40^ and graphene oxide.^37,40^ Graphene oxide in particular has been shown to be effective at forming mesopores within SAPO systems, as the more hydrophilic, oxidic surface interacts more strongly with the synthesis components than less oxidic carbon systems.^37,40^ Schmidt *et al.* were among the first to investigate the use of carbon templates to improve MTO lifetime for SAPO-34, and notably demonstrated the difference between including carbon nanoparticles (CNP) and carbon nanotubes (CNT).^36^ Despite greatly increasing the pore volume from 0.26 to 0.35 cm^3^ g^−1^, the inclusion of CNP did not improve the catalytic performance. This is attributed to the mesopores being strictly inside the catalyst particle, and as such not providing an extra surface for coke to form on.^36^ In contrast the CNT synthesised SAPO-34 greatly increased the external surface, extending the lifetime from 150 to 300 minutes.^36^ This suggests it is important to consider not just the size of the mesopores in HP systems, but also their shape, location, and composition.

The dehydration of bio-based ethanol to ethylene can offer a sustainable route to a vital plastic precursor. As such there is great interest in understanding the factors that dictate the activity of ethanol dehydration, such as pore-size, acid site strength and mesoporosity. Previous work has shown that SAPO-5 and SAPO-34 can readily activate ethanol and form diethyl ether, whereas ethylene formation requires stronger acid sites.^18,45,46^ Therefore, both the conversion and selectivity of this reaction are excellent indicators of solid-acid properties. In this case SAPO-5 was chosen to explore the versatility of hard-templating approaches to a wider range of SAPO systems, beyond the previously investigated SAPO-34 (ref. 33, 36, 38–40 and 42) and SAPO-11 systems.^47–49^ In this work, we will compare the activity for ethanol dehydration of a conventional microporous SAPO-5 (MP-SAPO-5) with similar HP-SAPO-5 systems synthesised with carbon nanoparticles (CNP-SAPO-5) and with carbon nanotubes (CNT-SAPO-5). Uniquely in this study, we will use a combination of small angle neutron scattering (SANS) and transmission electron microscopy (TEM) to understand hard templated SAPOs. Our findings will be correlated with catalytic activity measurements to understand the influence of mesoporosity on our systems.

## Experimental methods

### Synthesis

Spherical CNP (NIPex 160 IQ, from Orion Carbons), with a 20 nm diameter, and multi-walled carbon nanotubes (MWCNT) NC7000 from Nanocyl, with a 9.5 nm diameter and 1.5 μm length were used as carbon templates in this study. We adapted a recent SAPO-5 synthesis^50^ to incorporate the carbon templates, creating a robust synthesis protocol: 5.6 g of 85 wt% H_3_PO_4_ was diluted with 22.5 g of H_2_O and stirred. To this stirred solution 3.0 g of pseudo boehmite was added slowly. The suspension was stirred for 4 hours. After this 4.06 g of triethylamine was added dropwise with 1.55 g of 40 wt% Ludox AS-40 Colloidal Silica suspension in water. This was stirred for a further 2 hours. The carbon template (0.80 g of CNT or CNP) was added and stirred for another hour. The gel was then put into Teflon-liners and sonicated for 15 minutes, before being transferred into a sealed steel autoclave. The system was then heated at 200 °C for 24 hours in a preheated oven. On removal the samples were cooled and centrifuged thrice at 5000 rpm in a Heraues Megafuge 8 Centrifuge, equipped with a Thermo Scientific HIGHConic III Fixed Angle Rotor, with fresh deionised water each time. The as-synthesised samples were then dried overnight at 70 °C, before being calcined, under a 200 mL/min flow of air, at 600 °C for 16 hours, at a ramp rate of 2.5 °C min^−1^.

### SANS

SANS measurements were performed on the SANS2D small-angle diffractometer^51,52^ at the ISIS Neutron and Muon Source, Didcot, UK.^53^ This is a variable-geometry “white beam” time-of-flight instrument which utilizes neutrons with wavelengths, *λ*, between 1.75 to 12.5 Å. Data were simultaneously recorded on two, two-dimensional, position sensitive, neutron detectors situated at 5 and 12 m from the samples, offset slightly to either side of the transmitted beam, to simultaneously probe scattering vectors in the range ∼0.0015 < *Q* < 1.0 Å^−1^, or length scales ∼6 < *d* < 4000 Å, where:*Q* = (4π/*λ*)sin *θ* = 2π/*d*and 2*θ* is the scattering angle. The neutron beam incident on the samples was collimated to 6 mm in diameter. 0.15 g of dried SAPO-5 samples were densely packed into rectangular, 1 mm pathlength, synthetic quartz cuvettes (Starna type 1, Hellma type 100). An empty cuvette was also measured as background. Scattering data on each sample or background were accumulated for a total of 120 minutes to gather data of high statistical precision. Transmission (neutron absorption) data were accumulated for 10 minutes. Here SANS was chosen over SAXS due to the ability to probe hydrocarbon species within more electron-dense frameworks.^50^

Each ‘raw’ 2D data set was then corrected for the detector efficiencies and spatial linearity, sample transmission and background scattering, and reduced to 1D differential scattering cross-section data (∂*Σ*/∂*Ω vs. Q*) using the MantidWorkbench framework (version 6.3.0).^54,55^ These data were then placed on an absolute scale (cm^−1^) by comparison with the scattering from a standard sample (a solid blend of hydrogenous and perdeuterated polystyrene of known molecular weight) measured under the same instrument configuration in accordance with established procedures.^56^ In common with the accepted convention in SANS, we shall henceforth refer to (∂*Σ*/∂*Ω*) as ‘intensity’, *I*(*Q*). To derive meaningful structural information from the reduced data, as opposed to a fully quantitative structural refinement, optimised model-fitting was conducted using the SasView program (version 5.0.5).^57^ Further details of this are provided in the ESI.†

### Transmission Electron Microscopy (TEM)

For electron microscopy analysis, the samples were prepared by ultramicrotomy; initially the particles were imbedded in Epofix resin and the samples were left to harden overnight at 60 °C. Sections of 70 nm were then cut with a Reichert-Jung Ultracut E mictrotome with a Diatome Ultra 35° diamond knife. The sections were deposited on a 200 mesh holey carbon-coated Cu grid. The measurements were performed on a Thermo Fisher Scientific Talos F200X microscope at the Electron Microscopy Centre at Utrecht University, operated at 200 kV in TEM and HAADF-STEM mode.

### Catalysis

Ethanol dehydration catalysis was performed using a custom-built flow reactor provided by Cambridge Reactor Design. 0.3 g of calcined catalyst was used for each reaction, sieved between 300–500 μm. The sample was dried at 400 °C for 1 hour under a 25 mL min^−1^ flow of nitrogen prior to the reaction. Reactions were conducted at 230 °C, at which point the nitrogen flow was kept at 25 mL min^−1^, and a liquid flow of 3.13 μL min^−1^ of 10 vol% heptane (internal standard) in ethanol was flown (at a weight-hourly-space velocity; WHSV of 0.5 h^−1^). After 40 minutes, 250 μL of the vaporized output was injected as a gas into a PerkinElmer Clarus 460 GC with a Flame Ionized Detector, with a HP1 cross linked methylsiloxane (30 m × 0.32 mm × 1 mm film thickness) column. All results shown are the average of three consistent samples. Based on this we calculate the error of each measurement is ±3 mol%. The ethanol flow was also tested at 4.69, 6.25, 7.82, 9.38, 10.94 and 12.51 μL min^−1^ (corresponding to WHSV of 0.75, 1.0, 1.25, 1.5, 1.75 and 2.0 h^−1^), and resampled after 40 minutes as required.

Molar quantities of all observed molecules; ethanol, ethylene and diethyl ether, were calculated using calibrations with the heptane standard. Conversions and Yields were calculated using the equations below:
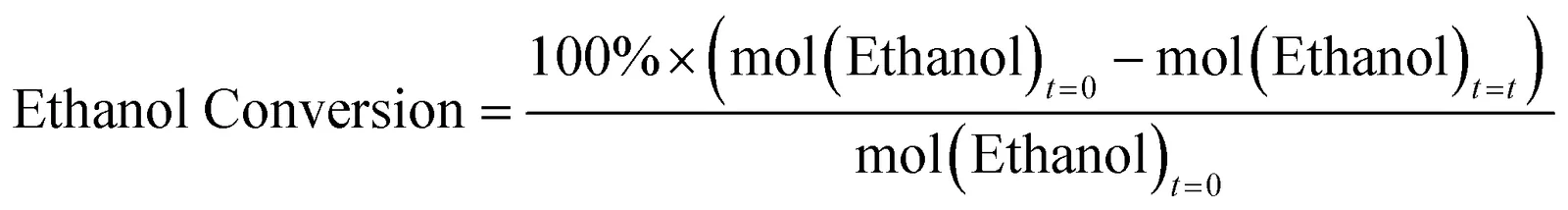

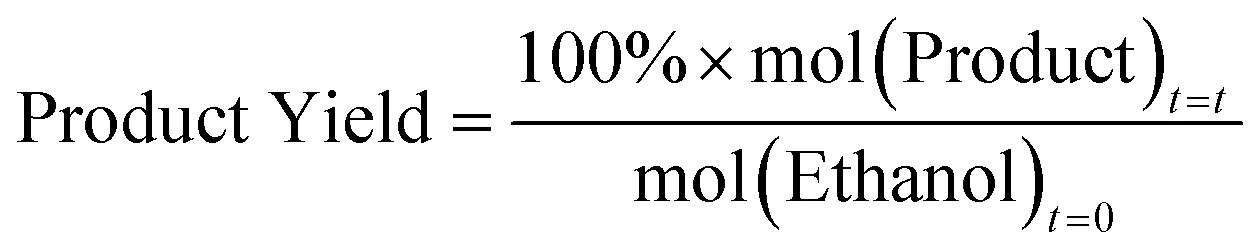


As these metrics are calculated with respect to the initial moles of ethanol, then the maximum expected product yield for diethyl ether is 50 mol%, given the reaction is: 2 ethanol → diethyl ether + H_2_O. This is done to allow simple comparisons of diethyl ether and ethylene on a molar basis.

Detailed experimental notes on X-ray diffraction (XRD), nitrogen physisorption (BET), solid state NMR (ssNMR), inductively coupled plasma (ICP), scanning electron microscopy (SEM), temperature programmed desorption (TPD) and elemental analysis (CHN) are given in the ESI.†

## Results and discussions

### Confirming the structural integrity

**Fig. 1:**
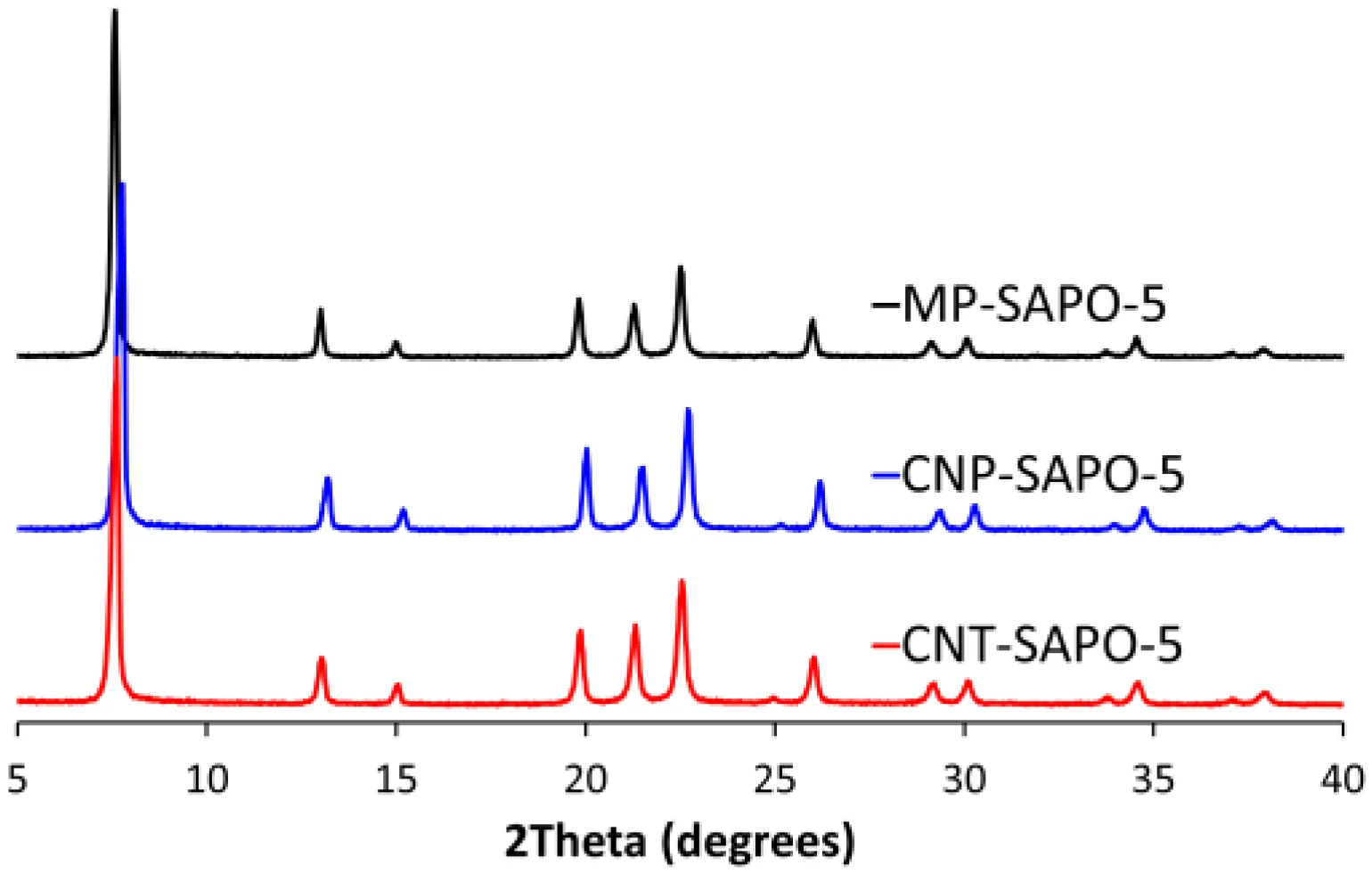
Powder XRD patterns of the three SAPO-5 species.

The powder X-ray diffraction (XRD) patterns of the calcined two carbon-based systems (CNP-SAPO-5 and CNT-SAPO-5) were compared with the calcined conventional microporous SAPO-5 (MP-SAPO-5; Fig. 1). All three systems were found to be crystalline, due to the sharpness of the Bragg peaks. Further only signals attributable to AlPO-5 framework (AFI framework code) were observed, suggesting that the systems exclusively formed the intended framework.^58^ There is some variation between the samples, notably the position of the 100 peak at ∼7.5° varies between the samples where the 2*θ* values change as MP-SAPO-5 < CNT-SAPO-5 < CNP-SAPO-5, which indicates a difference in lattice parameters in the HP systems. We also note that there is a difference in the relative intensity of the 210 peak (20.0°) compared to the 002 peak (21.3°) between the systems, with the 210 peak being more intense in the CNP-SAPO-5 system, and marginally more intense in MP-SAPO-5, whereas the 002 peak is clearly more intense in the CNT-SAPO-5 species. This suggests a subtle change in the structure factor between the three systems. Overall, all three systems were found to be crystalline and phase-pure SAPO-5.

The chemical composition of the three systems was determined using a combination of inductively coupled plasma (ICP) and elemental analysis (CHN). The Al and P loadings are consistent between the three samples, with the MP-SAPO-5 having slightly higher loadings of both (Table S1†).

In contrast the inclusion of the mesopore template into the synthesis has improved the Si uptake in the framework, with MP-SAPO-5 having 2.33 wt% Si, compared to 3.84 and 3.82 wt% Si in CNP-SAPO-5 and CNT-SAPO-5 respectively. This phenomenon has previously been reported in other HP-SAPO systems.^41,48^ In all cases there is no nitrogen present, confirming the complete removal of the microporous triethylamine template. There is minimal carbon remaining in all three systems (<1 wt%) however this is notably higher in the CNT-SAPO-5 system (0.5 wt%) than in the other two species, suggesting some of the CNT may not have been completely removed. Higher temperatures could be used to quantitatively remove the carbon; however, this may destabilise the framework. However, comparing the carbon content before and after calcination shows most of the carbon has been removed in all cases (Table S1†). We note that the values for elemental composition differ from our previous work, with the current MP-SAPO-5 having a greater amount of Si present (Table S1†).

**Fig. 2:**
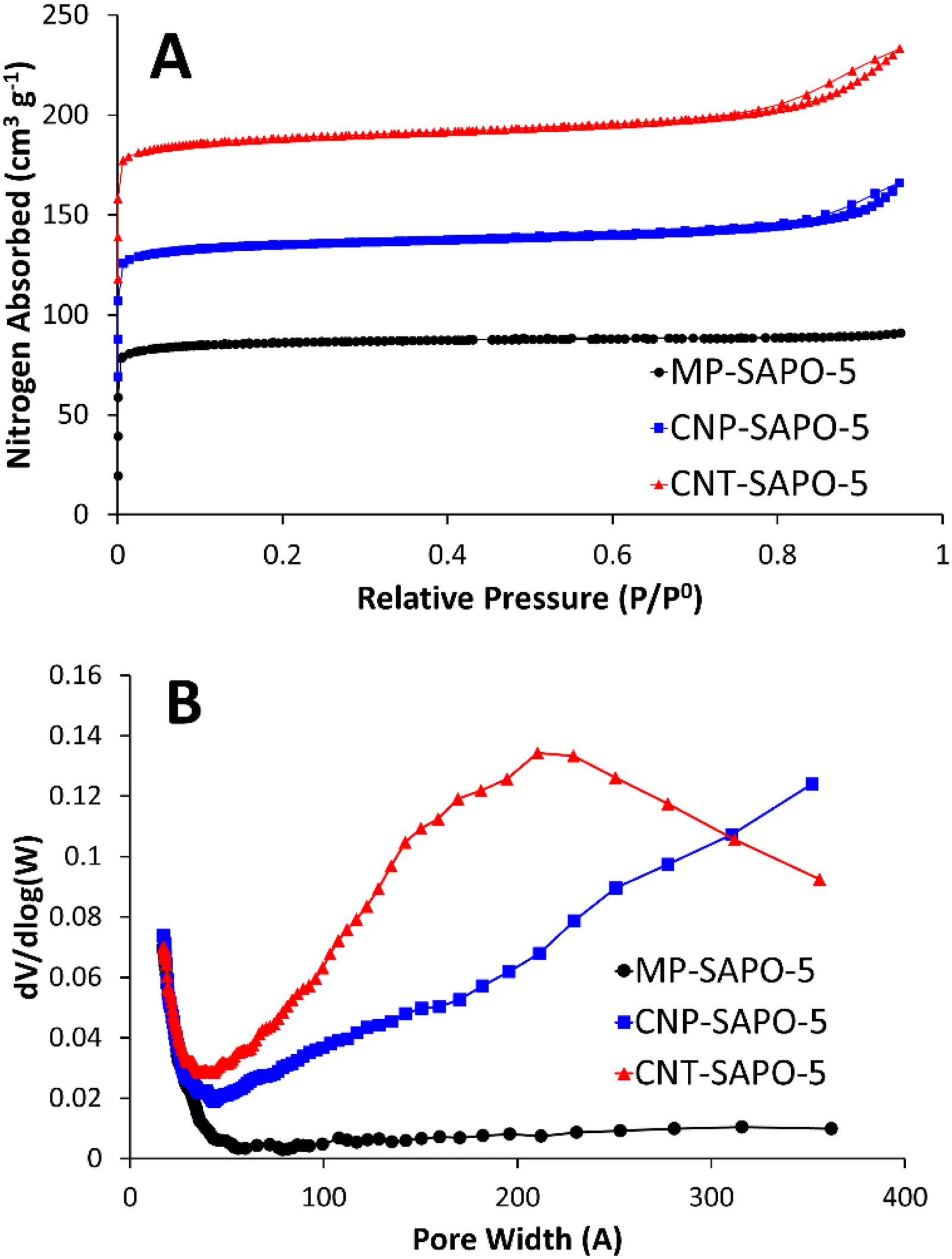
Showing the variation in porosity of the three calcined SAPO-5 systems, focusing on A) the physisorption isotherms (sequential data sets have been offset by 50 cm^3^ g^−1^ for ease of analysis) and B) the corresponding pore-size distributions.

Nitrogen physisorption was used to probe the porosity of the calcined samples (Fig. 2). The conventional MP-SAPO-5 presents a typical type-I isotherm (Fig. 2A), suggesting the system is microporous, as expected. Both CNP-SAPO-5 and CNT-SAPO-5 additionally show a type-IV hysteresis at relative pressures >0.7, associated with mesoporosity.^48^ Analysing the pore-distribution (Fig. 2B) of the systems confirms no significant mesoporosity was present in the MP-SAPO-5 system, whereas both CNP-SAPO-5 and CNT-SAPO-5 show features between 50–400 Å. CNP-SAPO-5 shows a differential pore volume increasing with pore size, beyond 350 Å. The CNP used were 200 Å in diameter, suggesting these mesoporous features could be induced by agglomerates of these primary particles. The CNT-SAPO-5 species shows a maximum value at a pore size of 210 Å, whereas the CNT used had a diameter of 95 Å, again possibly explained by clustering of the carbon template. Thus, the physisorption confirms that introducing carbon templates does indeed lead to mesoporosity. Comparing the surface areas and pore volumes (Table S2†) shows that the surface areas (262 to 274 m^2^ g^−1^) and micropore volumes (0.12 to 0.13 cm^3^ g^−1^) are similar for all three samples, and in good agreement with literature values of SAPO-5.^18^ However, the addition of carbon templates slightly increases the mesopore volume from 0.02 cm^3^ g^−1^ to 0.06 and 0.09 cm^3^ g^−1^ (MP-SAPO-5, to CNP-SAPO-5 and CNT-SAPO-5 respectively), again confirming that the inclusion of these carbon templates has induced mesoporosity.

Solid state NMR (ssNMR; Fig. S1†) was used to again confirm the integrity of the systems (^27^Al and ^31^P), and to probe the Si incorporation mechanism (^29^Si and ^1^H). The ^27^Al spectra (Fig. S1A†) shows all three systems have just one peak between 35 and 37 ppm, commonly attributed to the expected tetrahedral Al(iii)O_4_ species.^45,46,50^ Similarly, in the ^31^P spectra (Fig. S1B†) all three species show a single signal at −30 ppm, attributed to expected tetrahedral P(v)O_4_ species.^45,46,50^ The ^29^Si NMR (Fig. S1C†) can be used to probe the different silicon substitution methods into the AlPO backbone of the SAPO. In all cases the SAPO's primarily show a signal at either −94 or −97 ppm, attributed to silicon; Si(OAl)_3_(OSi) on the periphery of silicon islands.^45,46,50^ In all cases there is a slight signal at −91 ppm, attributed to isolated Si(OAl)_4_.^45,46,50^ The ^1^H NMR (Fig. S1D†) directly looks at the acid sites, with MP-SAPO-5 having a dominant signal at 4.6 ppm, attributed to Si(OH)Al species, with a smaller signal at 1.9 ppm due to silanol species.^59–61^ There is also a subtle signal at 3.6 ppm, attributed to Si(OH)Al protons in large unconfined channels or pores. The CNP-SAPO-5 and CNT-SAPO-5 species both show a greater quantity of silanol sites (1.0 ppm), and unconfined protons (3.6 ppm) than MP-SAPO-5.^59–61^ Likely the presence of mesopores leads to less confinement, and more surface defect sites. The CNP-SAPO-5 and CNT-SAPO-5 show different dominant signals with CNP-SAPO-5 presenting at 5.5 ppm, and CNT-SAPO-5 showing at 4.6 ppm. Both of these have previously been attributed to Si(OH)Al species.^59–61^ Overall ssNMR shows subtle differences between the nature of the silicon and proton sites.

Ammonia-temperature programmed desorption (NH_3_-TPD) probed the influence of the different carbon templates on acidity (Fig. S2 and Table S3†). CNP-SAPO-5 has a lower total acidity to MP-SAPO-5 (29 400 and 35 400 mV s g^−1^, respectively), whilst also having far fewer stronger acid sites (550 to 600 °C; 19 and 8%, respectively). In comparison, CNT-SAPO-5 has more acid sites than both CNP-SAPO-5 and MP-SAPO-5 with 42 100 mV s g^−1^. Comparing the distribution of acid sites shows that despite CNT-SAPO-5 having more acid sites than MP-SAPO-5, the acid site strength distribution is quite similar (Table S3†).

Scanning electron microscopy (SEM) explored the particle size, shape, and uniformity of the three samples (Fig. S3†). MP-SAPO-5 is composed of aggregates of small (1 × 2 μm) hexagonal crystals, as previously observed, and in good agreement with its crystallographic space group (Fig. S3A–D†).^62^ The CNP-SAPO-5 forms particles of similar size and shape (Fig. S3E–H†) to MP-SAPO-5. In contrast the CNT-SAPO-5 shows some hexagonal crystals (Fig. S3I–L†), however these are highly aggregated, and less uniform than the other two SAPO species.

**Fig. 3:**
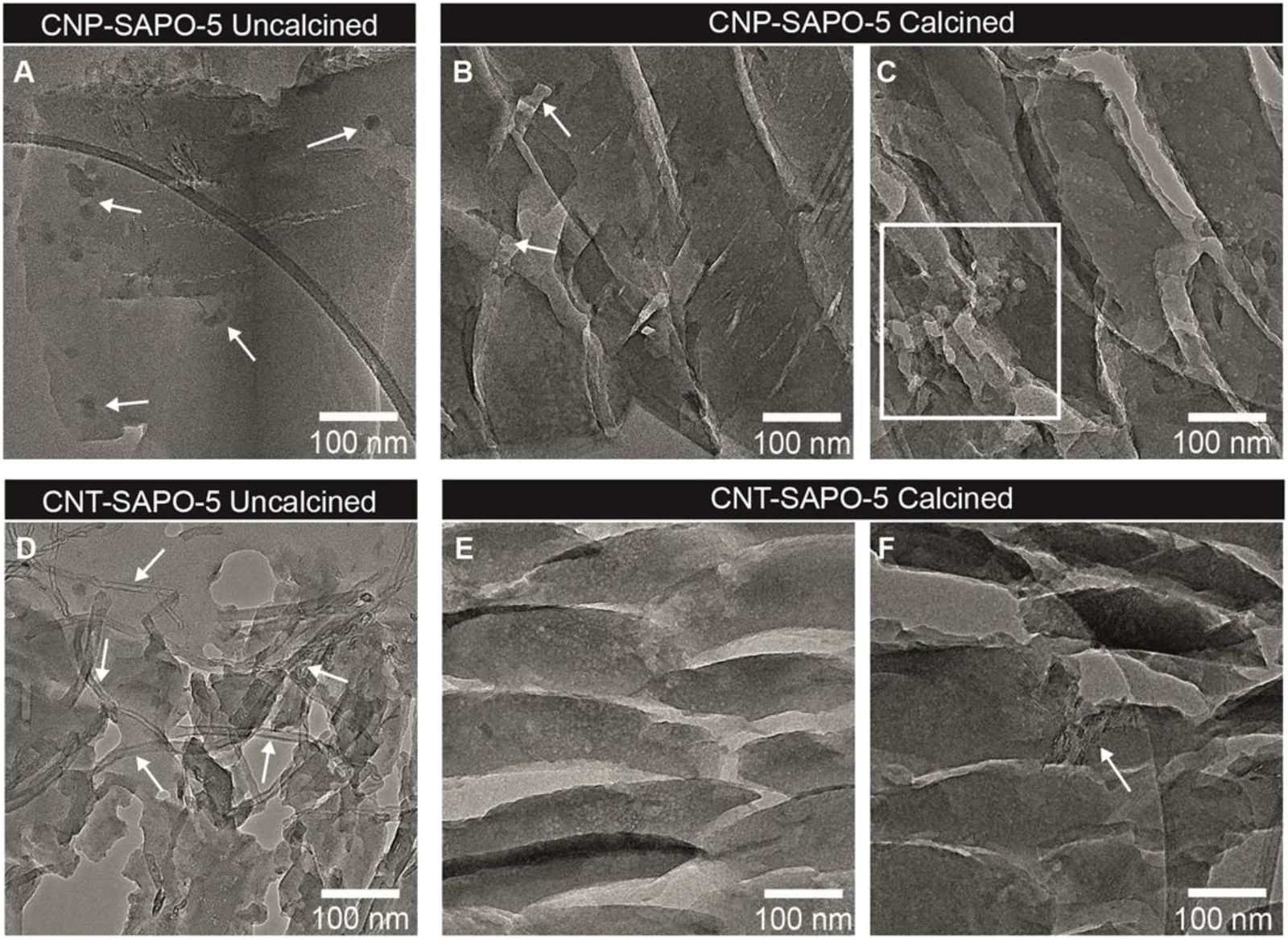
TEM images of A) uncalcined CNP-SAPO-5, where the white arrows indicate the presence of CNP, B and C) calcined CNP-SAPO-5, where the arrows indicate the presence of pores due to the removal of single CNP and the white box highlights the presence of pores due to removal of a cluster of CNPs. D) Uncalcined CNT-SAPO-5, clearly showing the presence of CNT. E and F) Calcined CNT-SAPO-5, where the white arrow indicates the remainder of some CNT.

Transmission electron microscopy (TEM) analysis was performed to visualize the three SAPO-5 samples. Using ultramicrotomy, slices of 70 nm were cut, which were imaged in the microscope. This enhanced the visibility of the three SAPO-5 materials to determine the presence of mesopores within the crystals (Fig. 3 and S4†). Both the uncalcined and calcined systems were analysed, to locate the carbon templates. The MP-SAPO-5 species (Fig. S4†) is highly crystalline both uncalcined (Fig. S4A†) and calcined (Fig. S4D†). We note the cracks in the sample are due to the microtoming sample preparation. The uncalcined system appears very smooth (Fig. S4B†), with increased roughness on calcination (Fig. S4E†). We noticed the presence of small 1–2 nm nanoparticles in both the uncalcined (Fig. S4C†) and calcined (Fig. S4F†) sample, although their nature was not identified these most likely consisted of very small quantities of more densely packed silica or alumina, as no metals were observed with ICP analysis. For MP-SAPO-5, there were no obvious signs of higher-level porosity, in good agreement with the BET data (Fig. 1 and Table S2†). TEM images of uncalcined CNP-SAPO-5 clearly show the presence of the ∼20 nm diameter CNP spheres within the smooth SAPO particles, indicated with arrows in Fig. 3A. These did not appear to be uniformly distributed throughout the sample. Some seemed to aggregate, whereas in other locations of the SAPO-5 the CNP were absent. On calcination of the CNP-SAPO-5, some lighter regions were observed in the TEM images (Fig. 3B and C). Although it is impossible to fully exclude the formation of artifacts due to the preparation of the SAPO-5 structure, causing this observation, these regions were approximately 18–25 nm in size and similar in shape to the CNP. This suggests that these could be mesopores formed due to the removal of either single (Fig. 3B) or aggregated (Fig. 3C) CNP during calcination. Although tomography could be used to further study if these were indeed mesopores, this was beyond the scope of the current study. The uncalcined CNT-SAPO-5 system (Fig. 3D) clearly shows the presence of the CNT running through the sample, however from the TEM analysis it is not possible to establish if this is within the framework, or simply on the external surface. Less magnified Images of CNT-SAPO-5 (Fig. S5†) show that the CNT SAPO-5 does not break in a similar fashion as the SAPO-5 during ultramicrotomy, suggesting the CNT is likely affecting the external surface, as seen by CNT spanning voids between SAPO-5 crystals (Fig. S5†). On calcination, the surface of the particles appears smoother, with, contrary to the removal of CNP, barely any indication of the formation of mesopores due to the removal of CNT (Fig. 3E). There was some (Fig. 3F), but overall, very limited evidence the CNT remaining. This matched the elemental analysis, that most carbon was removed from CNT-SAPO-5 during calcination. This suggests that whilst CNT leads to external porosity in CNT-SAPO-5, it may not form internal mesopores within the CNT-SAPO-5 particle, and that the differences in porosity may be due to other factors, such as the mere inclusion of CNT in the synthesis, or surface roughness.

### Interpreting the porosity

**Fig. 4:**
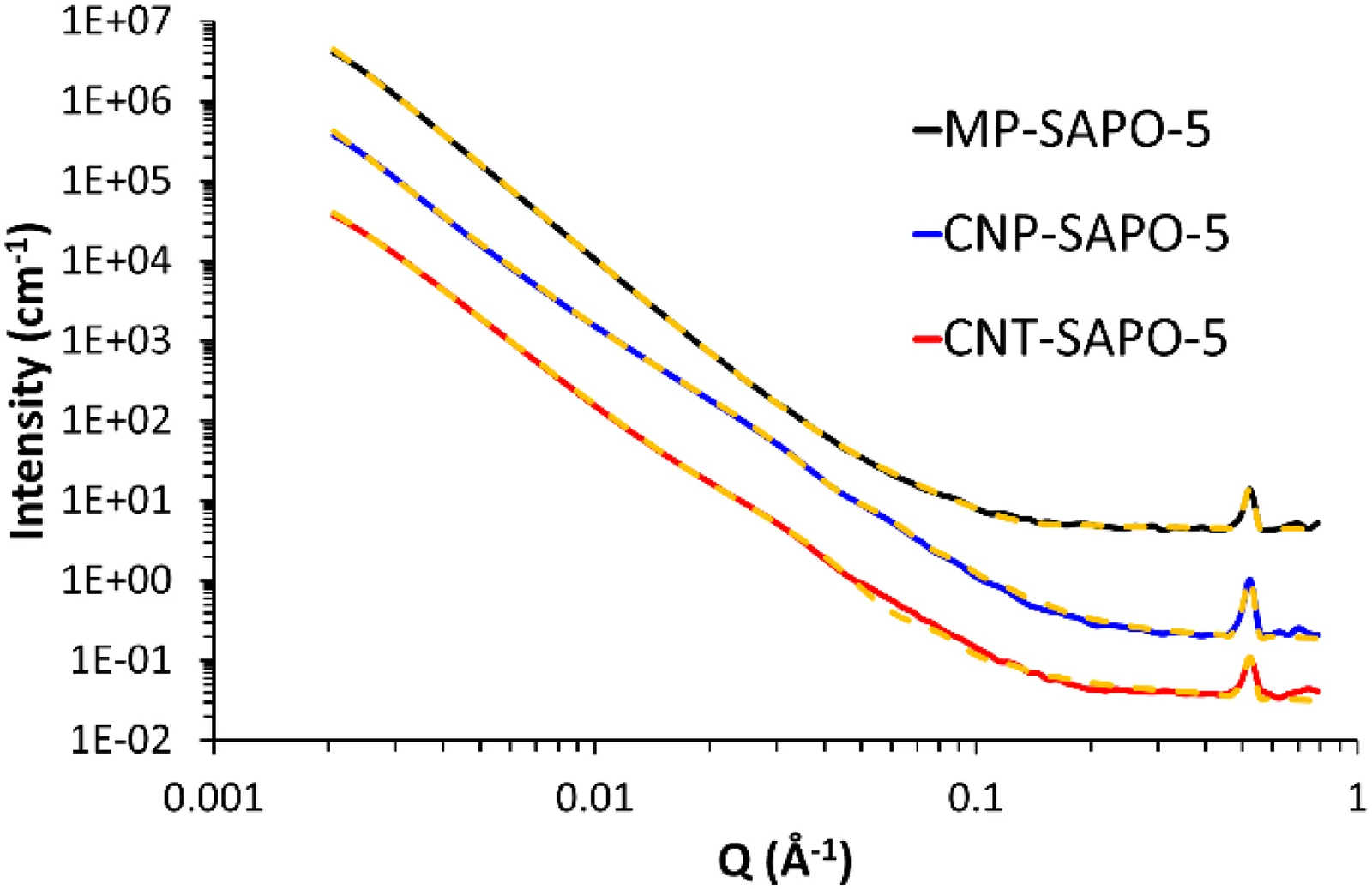
SANS data from the 3 SAPO-5 systems (solid lines) with their final fits (yellow dashed lines). The data for MP-SAPO-5 and CNP-SAPO-5 are displaced by factors of 100 and 10, respectively, for clarity. Derived structural parameters are summarised in Tables 1 and S5.† Fitting residuals are shown in Fig. S8.† For a description of the fitting model see the main text.

We employed small angle neutron scattering (SANS, Fig. 4) to probe the porosity further, to complement and compare with our TEM and BET findings. For a brief introduction to the SANS technique, and its application to hierarchically porous framework systems, we refer the reader to our previous paper and the references therein.^50^ The scattering from all three systems here is characterised by a quasi-power law region at lower *Q* (larger *d*) values, decaying as *Q*^−*n*^ where *n* ∼ 3, and arising from intrinsically rough solid–air interfaces, but modulated by geometric contributions from the shapes of the pores. At higher *Q* (smaller *d*) values, the scattering is dominated by short-range ordering superimposed on a background signal. The peak evident at *Q* ∼ 0.53 Å^−1^ is easily shown to be that present at 2*θ* ∼ 7.5° in Fig. 1 for Cu_Kα_ radiation. To interpret the SANS, we extract both n and the constituent length scales using the approach previously employed. This involves least-squares fitting the SANS data to a weighted linear combination of functions (‘the model’) describing the underlying structure. The models used here are in fact a slight simplification as the SANS2D instrument could not access the 110 peak in the configuration used. The current SANS data is compared to our previous work in Fig. S6,† with associated discussions.

**Table 1:** Summary of the key parameters from fitting the SANS data from the three SAPO-5 systems with a model combining a power law, fractal aggregate, sphere and Gaussian peak. For the in-depth fitting parameters and uncertainties, see Tables S5 and S6 in the ESI.† For each parameter, the first row is when only allowing the micropores to exhibit size dispersity. The second row is allowing both micropores and mesopores to exhibit size dispersity

Parameter	MP-SAPO-5	CNP-SAPO-5	CNT-SAPO-5
Relative component weightings^a^	1 : 2902 : 77	1 : 1 700 288 : 37 175	1 : 4 030 226 : 226 700
	1 : 3295 : 126	1 : 4 758 064 : 140 322	1 : 62 780 : 8856
Relative micropore weightings^b^	1.0	2.3	1.2
	1.0	2.3	1.1
Relative mesopore weightings^b^	1.0	1.9	2.6
	1.0	1.7	3.9
Fractal dimension	3.0	2.9	2.9
	3.0	2.9	2.9
Ave. micropore width (Å)^c^	3.8	2.8	5.8
	2.2	2.0	6.1
Ave. mesopore width (Å)^c^	60	204	148
	24	160	36

a Based on scale factors reported in Tables S5 and S6.† Power law (A) : fractal (B) : sphere (C).

b Based on scale factors reported in Tables S5 and S6.† MP-SAPO-5 : CNP-SAPO-5 : CNT-SAPO-5.

c 2 × equivalent spherical radius.

The final fitting model comprised a power law, a fractal aggregate of spherical objects (to account for the micropores), a spherical form factor (to account for the mesopores), a Gaussian peak function, and a flat background.

As the elemental analysis data (Table S1†) shows that the carbon from the templates has been almost completely removed we did not try to account for it.

During the fitting the neutron scattering length density (SLD) of the pores and the matrix were fixed at their calculated values of 0 (air) and 4.1 × 10^−6^ Å^−2^, respectively. And, guided by our previous work, the spherical building blocks in the fractal component were ascribed some size dispersity. To demonstrate the sensitivity of this approach, Table S4 and Fig. S7† show the results of fitting the model without the spherical form factor component which represents mesopores. As expected, a reasonable fit was achieved for MP-SAPO-5, whereas this model was not able to describe the CNP-SAPO-5 and CNT-SAPO-5 systems anywhere near as successfully (shown most clearly in Fig. S7D†). This illustrates that other structural components are required to better describe the hard-templated systems.

Table S5 and Fig. S8† demonstrate the impact of including the spherical form factor component (with no-polydispersity, representing variations in size) to represent mesoporosity. All both the CNP-SAPO-5 and CNT-SAPO-5 fits are dramatically improved (with the fitting for MP-SAPO-5 included for completeness). Whilst it is tempting to simply assign this to the inclusion of two additional adjustable parameters in the model, the minimisation of *χ*^2^ during the fitting optimisation takes this into account (*i.e.*, it uses a reduced-*χ*^2^). Thus, the reduction in observed *χ*^2^ (CNP-SAPO-5: 211 → 48; CNT-SAPO-5: 99 → 35) is statistically significant. This model is still not perfect, however, with some notable discrepancies at low-*Q*, where variations in particle size, roughness, *etc.*, are a factor.

Table 1 shows a summary of the key structural parameters derived from the SANS data. The individual component contributions to each fit are shown in Fig. S9.† The fractal dimensions confirm the porous nature of all three systems, and there is more porosity in the hard-templated systems, supporting the BET measurements. Allowing the width of the spherical component to vary (introducing polydispersity) shows a slight variation in the CNP-SAPO-5 pore width (204 to 160 Å), but a significant change in CNT-SAPO-5 pore width (148 to 36 Å). The minimal variation in the CNP-SAPO-5 pore width suggests these mesopores are genuinely present inside the system, as seen through TEM. However, the significant change in CNT-SAPO-5 suggests that this model is not well suited to explaining the mesoporosity in this system, further emphasising that CNT were not as successful as incorporating into the system, and therefore did not result in defined mesopores. Similarly, this data also supports the finding that CNP-SAPO-5 has larger mesopores than CNT-SAPO-5 as per the physisorption values (Fig. 2). Quantitative agreement is not to be expected due to differences in how the two techniques account for the precise pore-size distributions, and because SANS will measure closed porosity too; nonetheless, the figures reported here and in Fig. 2B are within a factor of two. There are some subtle differences in the parameters depending on how pore size dispersity is accounted for, but the trends are the same. We also note a reasonable correlation between the relative mesopore weightings above and the relative mesopore volumes in Table S2† (respectively, 0.02, 0.06, and 0.09 cm^3^ g^−1^; a ratio of 1 : 3 : 4.5), which is also seen in the contributions from the sphere model (Fig. S9†). To conclude, we note that our findings on CNT-SAPO-5, and the findings of others, have suggested that more complex mesoporous structures are formed using CNT-templated systems than the interpretation here allows for.^18^ However CNP-SAPO-5 has formed defined mesopores.

### Catalytic performance

**Fig. 5:**
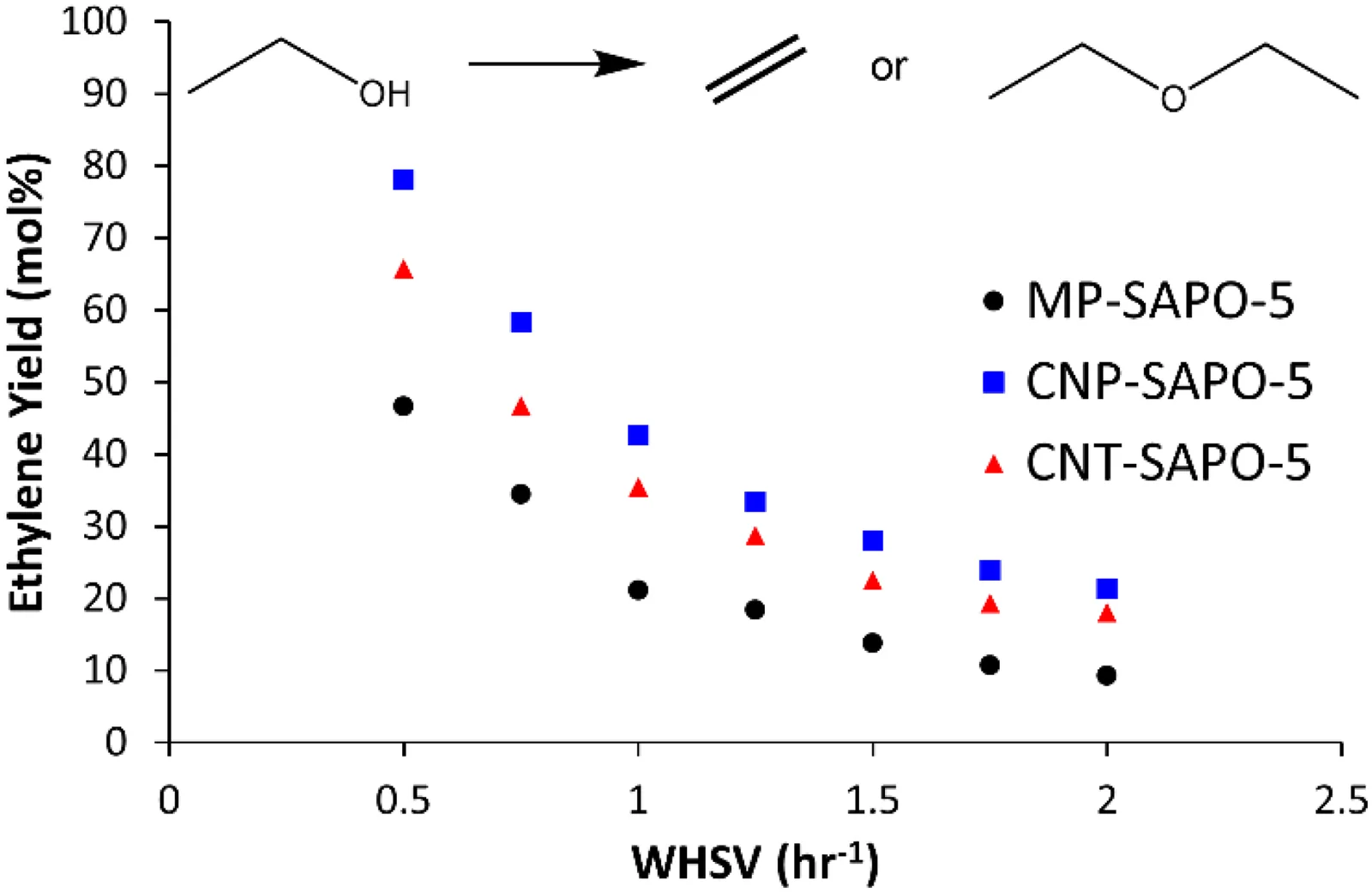
Catalytic data for ethanol dehydration with the three SAPO-5 systems showing the ethylene yield. Conditions: 230 °C, 25 mL min^−1^ N_2_ carrier gas, liquid feedstock of 10% heptane (internal standard) in ethanol, liquid flow varied from 3.13 to 12.51 μL min^−1^ as the WHSV varies from 0.5 to 2.0 h^−1^. Errors are not shown but are calculated as ±3 mol% based on multiple injections.

The three different SAPO-5 species were tested in the ethanol dehydration reaction at 230 °C to assess the influence of the added mesoporosity (Fig. 5 and S10 and Table S7†).^18^ All three samples show high ethanol conversions (between 95 and 85 mol%), which show little variation with temperature or WHSV (Fig. S10A and Table S7†). This suggests that the system is capable of fully converting the ethanol in this regime, and that deviation from complete conversion is likely due to packing inefficiencies. As discussed previously, ethanol conversion is not a sensitive parameter for comparing SAPO catalysts. Instead, the yields, and relative formation of ethylene and diethyl ether is considered more diagnostic, as this is known to be the more challenging transformation. However notable changes are seen in the selectivity (and therefore the yield) of diethyl ether and ethylene between the systems.

Both CNP-SAPO-5 and CNT-SAPO-5 show improved ethylene yields, at all WHSV, compared to the conventional MP-SAPO-5 (Fig. 5 and Table S7†). As the WHSV increases from 0.5 to 2.0 h^−1^ the ethylene yield (and selectivity) decreases for both systems, whilst the diethyl ether yield (and selectivity) increases. This suggests that either minimising contact time, or maximising ethanol concentration, favours ether formation over the alkene, in line with previous work,^18,45,46^ which suggested ethylene forms from a diethyl ether intermediate, and not directly from ethanol.

At a WHSV of 0.5 h^−1^ MP-SAPO-5 achieved an ethylene yield of 46.7 mol%, whereas the CNP-SAPO-5 and CNT-SAPO-5 systems achieved 78.2 and 65.8 mol%, respectively, under identical conditions; a notable improvement for the hierarchical systems over the conventional system. This improvement cannot be attributed to acidity, as CNP-SAPO-5 has slightly fewer and weaker acid sites than MP-SAPO-5. Instead, the most likely cause of this improvement is the added mesoporosity. This could be the result of the mesopores improving pore diffusion, allowing diethyl ether to encounter a greater number of acid sites.^40–42^ In both cases, the likelihood of an interaction between diethyl ether and an acid site is maximised. Our recent theoretical work explored the possibility of ethylene being formed either directly from ethanol, or through an alkoxy site.^18^ If this pathway were playing a notable role, then improved pore diffusion would result in ethanol being spread more evenly throughout a catalyst particle, reducing ethanol–ethanol interactions, and therefore favouring a monomolecular route to ethylene, and not diethyl ether. In CNT-SAPO-5 species, the number of acid sites is higher than MP-SAPO-5, which is likely a factor in the improved ethylene yields of CNT-SAPO-5 compared to MP-SAPO-5. Similarly, we note that the pore diameters, as determined by both BET (Fig. 2) and SANS (Fig. 4, Tables 1 and S5 and S6†) followed the trend of CNP-SAPO-5 > CNT-SAPO-5 > MP-SAPO-5, which correlates with the trend in ethylene yield we see here (Fig. 5 and Table S7†). As such combining this data allows us to conclude that the addition of mesoporosity strongly correlates to the improved catalytic activity of SAPO-5 for ethanol dehydration. We note that the catalytic findings in this work, using carbon templates as a hard template to form a HP-SAPO-5, differ significantly from previous work on using DMOD (dimethyloctadecyl[3-(trimethoxysilyl)propyl]ammonium chloride) as a soft template to form a HP-SAPO-5.^50^ Further, the findings here, of CNT improving catalyst yield of SAPO-5, are in contrast to other works, where adding CNT to SAPO-34 did not improve catalytic activity.^36^ This is not surprising as different SAPO materials will have different synthetic protocols, gel ratios, pH *etc.* As such modifications to one material, may prompt different effects in other materials.

In this work, we have chosen carbon as a hard-template, which has resulted in HP-SAPO-5 systems with improved ethanol conversion and improved ethylene yields. The carbon template, as shown from elemental analysis (Table S1†) left minimal residue in the mesopores, allowing them to be free, open and able to aid pore-diffusion, which has clearly benefitted the reactivity. This hypothesis, whilst clearly supported by our experimental findings, could be investigated further using spatially resolved spectroscopic techniques, such as infrared, like those performed on methanol-to-hydrocarbon systems,^63,64^ to observe the ingress and egress of the reaction components throughout the MP-SAPO-5, and different HP-SAPO-5 systems. Overall, this finding highlights the importance of careful selection of mesopore template for forming hierarchical solid acid catalysts, and how one can induce different catalytic behaviours with different templates.

## Conclusion

In this work we have adapted our synthetic protocol for microporous, conventional SAPO-5 to use carbon-based templates. This allowed us to study three SAPO-5 systems based on these methods, the conventional microporous SAPO-5 (MP-SAPO-5), SAPO-5 synthesised with spherical carbon nanoparticles (CNP-SAPO-5) and with multiwalled carbon nanotubes (CNT-SAPO-5). The structural integrity of these systems was confirmed using a variety of techniques (XRD, ssNMR, elemental analysis, NH_3_-TPD and SEM), confirming that the intended framework had formed, and highlighted the comparable acidic properties of the systems. Nitrogen physisorption showed the differing influence of the two carbon templates. Whilst both templates led to higher-level porosity, the CNP directly formed mesopores, due to the carbon template being incorporated into the framework, however the CNT did not. This reinforced previous literature findings that the size and shape of the hard template is an important factor in determining the final shape and size of the mesopores. Most notably a combination of TEM images and SANS measurements were used to fully delve into the different mesopore formation routes from the two carbon nanotemplates. By using a carbon nanotemplates, the entirety of the carbon can be removed on calcination, leaving open and available mesopores for improved pore diffusion, and catalytic activity. These new findings emphasise the importance of carefully chosen synthesis protocols for forming hierarchical materials, towards optimised solid acid catalysts.

## Author contributions

M. E. P. wrote the manuscript, performed materials synthesis, characterisation, experimental design and conceptualisation, and catalytic testing experiments. E. B. M. collected SEM images. N. L. V. and J. D. M. performed TEM analysis. L. J. A. helped design and perform the NH_3_-TPD experiments. S. M. K. performed and helped analysis the SANS data. M. C. performed and helped analyse the solid-state NMR data. P. E. d. J., B. D. V. and R. R. helped with funding acquisition, supervision, and manuscript editing. All the authors commented on the manuscript.

## Conflicts of interest

The authors declare that this study received funding from TotalEnergies OneTech Belgium. The funder had the following involvement in the study: discussion and interpretation of data, as well as the decision to submit and support in writing the publication.

## Supplementary Material

LF-001-D4LF00230J-s001

## Data Availability

Data for this article, including X-ray diffraction, N_2_ physisorption, solid state nuclear magnetic resonance, NH_3_-temperature programmed desorption data and small angle neutron scattering spectra are available at Open Science Framework at https://doi.org/10.17605/OSF.IO/UM3QX.
