# Supplementary material for: Exploring the influence of mesoporosity in hard carbon-templated hierarchical SAPO-5 for ethanol dehydration
Source: RSC Appl Interfaces. 2024 Aug 8;1(6):1360–70. doi: 10.1039/d4lf00230j (PMC13591374; doi:10.1039/d4lf00230j)
Supplement: Supplementary file 1 [file LF-001-D4LF00230J-s001.pdf]

Supplementary Information (SI) for RSC Applied Interfaces.
This journal is © The Royal Society of Chemistry 2024

SUPPLEMENTARY INFORMATION

Exploring the influence of mesoporosity in hard carbon-templated hierarchical SAPO-5 for ethanol dehydration

M. E. Potter[a,b,c]*, E. B. McShane[c], N. L. Visser[d], J. D. Meeldijk[d], L. J. Allen[a,b], S. M. King[e], M. Carravetta[c], P. E. de Jongh[d], B. D. Vandegehuchte[f] and R. Raja[c]

a. UK CatalysisHub, Research Complex at Harwell, Rutherford Appleton Laboratory, Didcot, OX11 0FA, UK.

b. Chemistry Department, University College London, 20 Gordon Street, London, WC1H 0AJ, UK.

c. School of Chemistry, University of Southampton, Highfield Campus, Southampton, SO17 1BJ, UK.

d. Materials Chemistry and Catalysis, Debye Institute for Nanomaterials Science, Utrecht University, Universiteitsweg 99, 3584 CG, Utrecht, The Netherlands.

e. ISIS Neutron and Muon Source, STFC Rutherford Appleton Laboratory, Harwell Campus, Didcot, OX11 0QX, UK

f. TotalEnergies, OneTech Belgium, Zone Industrielle Feluy C, B-7181 Seneffe, Belgium.

*MEP = mep61@bath.ac.uk

Contents

Experimental details 2
Structural characterisation data 3
Chemical composition 3
Nitrogen physisorption metrics 3
ssNMR spectra 3
NH_3-TPD results 5
Scanning electron microscopy (SEM) images 6
Transmission electron microscopy (TEM) images 8
Comparison of MP-SAPO-5 Morphology 9
Small angle neutron scattering (SANS) data fitting 10
Power law – Fractal – Gaussian Peak model 10
Power law – Fractal – Monodisperse Sphere – Gaussian Peak Model 12
Power law – Fractal – Polydisperse Sphere – Gaussian Peak Model 17
Descriptions of model components 18
Catalytic ethanol dehydration data 19
References 20

Experimental details

Powder Diffraction (XRD): Powder X-ray diffraction was per-formed on a Bruker D2 Phaser diffractometer using a 1D LYNXEYE detector and 0.6 mm slits, with Cu $K_{\alpha1}$ radiation. Patterns were run over a 2θ range of 5 to 40 ° with a scan speed of 3 ° min^{-1} and an increment of 0.01 °. Powdered samples were placed into a plastic holder with roughly 3 mm thickness.

Inductively Coupled Plasma (ICP): ICP was performed by MEDAC *via* their commercial mail-in service. For further details see: https://medacltd.com/services-view/metals-icp/.

Elemental Analysis (CHN): CHN was performed by London Metropolitan University *via* their commercial mail-in service. For further details see: https://www.londonmet.ac.uk/services-and-facilities/elemental-analysis-service/.

Nitrogen Physisorption (BET): BET surface area measurements were performed at 77 K, on a sample dried under 20 mTorr of vacuum at 120 °C overnight. Analysis was per-formed on a Micromeritics Gemini 2375 surface area analyser. Surface area was calculated using the BET model,[1] while the pore width distribution was calculated with the BJH model, which assumed cylindrical pores.[2] The "total pore volume" was calculated by converting the volume of gaseous nitrogen absorbed to the volume of liquid nitrogen adsorbed, using a conversion factor of 647. The BJH distribution plot[2] showed the variation in pore volume with respect to pore size, the total pore volume calculated for pores above 20 Å was taken as the "mesopore pore volume". The difference between the "total pore volume" and "mesopore pore volume" was calculated as being the "micropore pore volume".

Solid State Nuclear Magnetic Resonance (ssNMR): Samples were packed into 4 mm pencil-style zirconium oxide rotors and acquired at a spinning rate of 8 kHz using a 4 mm RevolutionNMR probe. ^{1}H, ^{27}Al, and ^{31}P spectra were acquired in triple resonance mode, and ^{29}Si in double resonance on a Bruker Avance Neo widebore Spectrometer, using a 9.4 T field, in air. The sample ^{1}H, ^{27}Al, and ^{31}P, and ^{29}Si T_1 relaxation time was assessed using a saturation recovery pulse technique, which was used for the respective direct acquisitions. Specifically the following conditions were used:

^{27}Al NMR: 16 scans, pulse delay of 7 seconds, short pulse excitation
^{31}P NMR: 4 scans, pulse delay of between 30 and 50 seconds
^{29}Si NMR: 4096 scans, pulse delay of 31 seconds, contact time of 6 milliseconds
^{1}H NMR: 8 scans, pulse delay of 3 seconds

Scanning Electron Microscopy (SEM): SEM images were acquired at the Southampton Biomedical Imaging Unit, using an FEI Quanta 250 FEG scanning electron microscope. Samples were sputter coated with platinum prior to imaging.

Transmission Electron Microscopy (TEM): TEM images were acquired at the Electron Microscopy Centre of the Utrecht University using a Thermo Fisher Scientific TalosF200X electron microscope

Temperature Programmed Desorption (TPD): NH_3-TPD was performed at the Research Complex at Harwell, as part of the UK Catalysis Hub, on a Quantachrome ChemBET Pulsar TPR/TPD Automated Chemisorption Analyser. 0.2 g of pelletised and sieved catalyst (150 – 425 µm) was used for each analysis. The sample was first recalcined at 550 °C for 2 hours, ramping at 10 °C min^{-1}, under a 30 mL min^{-1} flow of 20 % O_2 in N_2. The system was then cooled to 150 °C, and held for 2 hours whilst under a flow of 30 mL min^{-1} of 5% NH_3 in He. The flow was then changed to 30 mL min^{-1} of He, and held at 150 °C for a further 2 hours. After which the system was then heated to 600 °C at a rate of 5 °C min^{-1} and held for 1 hour. The data was then normalised and background subtracted to give values as a function of signal (mV g^{-1}) versus temperature (°C).

Structural characterisation data

Chemical composition

Table S1: Showing the variation in elemental composition of the three calcined SAPO-5 systems "Calc", contrasting the carbon and nitrogen content with the as-synthesised, uncalcined systems "AS".

Sample	Al (wt%)	P (wt%)	Si (wt%)	(mol P + mol Si) / mol Al	C (wt%)	N (wt%)
**MP-SAPO-5 Calc**	18.0	20.0	2.33	1.09	0.1	0.0
**MP-SAPO-5 AS**					5.7	1.1
**CNP-SAPO-5 Calc**	17.0	18.1	3.84	1.14	0.0	0.0
**CNP-SAPO-5 AS**					18.0	1.2
**CNT-SAPO-5 Calc**	17.1	18.2	3.82	1.14	0.5	0.0
**CNT-SAPO-5 AS**					14.7	0.7

Nitrogen physisorption metrics

Table S2: Nitrogen physisorption values for the three SAPO-5 systems.

Sample	BET Surface Area (m^2 g^{-1})	Total Pore Volume (cm^3 g^{-1})	Micropore Volume (cm^3 g^{-1})	Mesopore Volume (cm^3 g^{-1})
**MP-SAPO-5**	263	0.14	0.13	0.02
**CNP-SAPO-5**	262	0.18	0.12	0.06
**CNT-SAPO-5**	274	0.21	0.12	0.09

ssNMR spectra

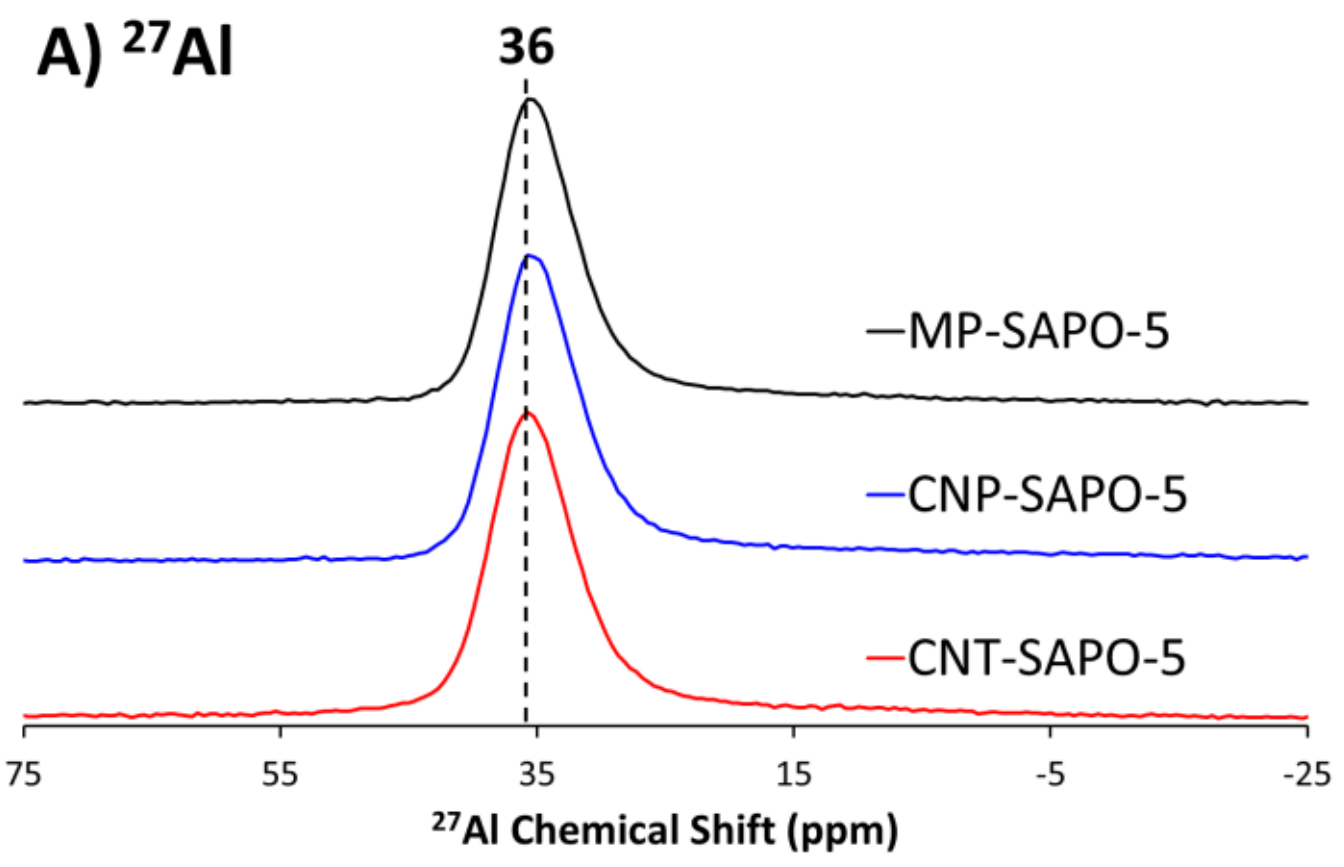

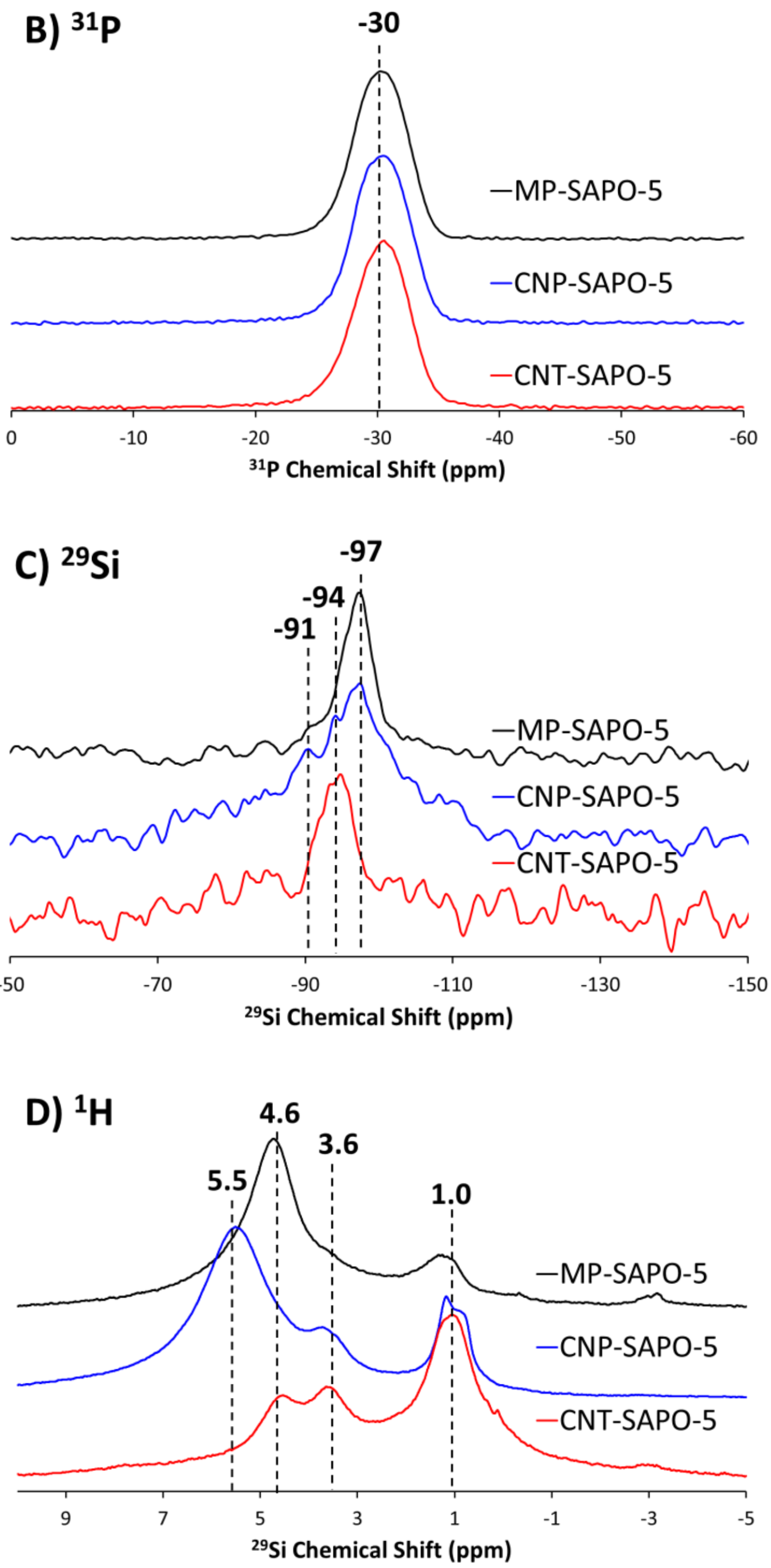

Fig. S1: ssNMR of the three SAPO-5 species, focussing on A) ^{27}Al, B)^{31}P, C) ^{29}Si and D) ^{1}H nuclei.

NH_3-TPD results

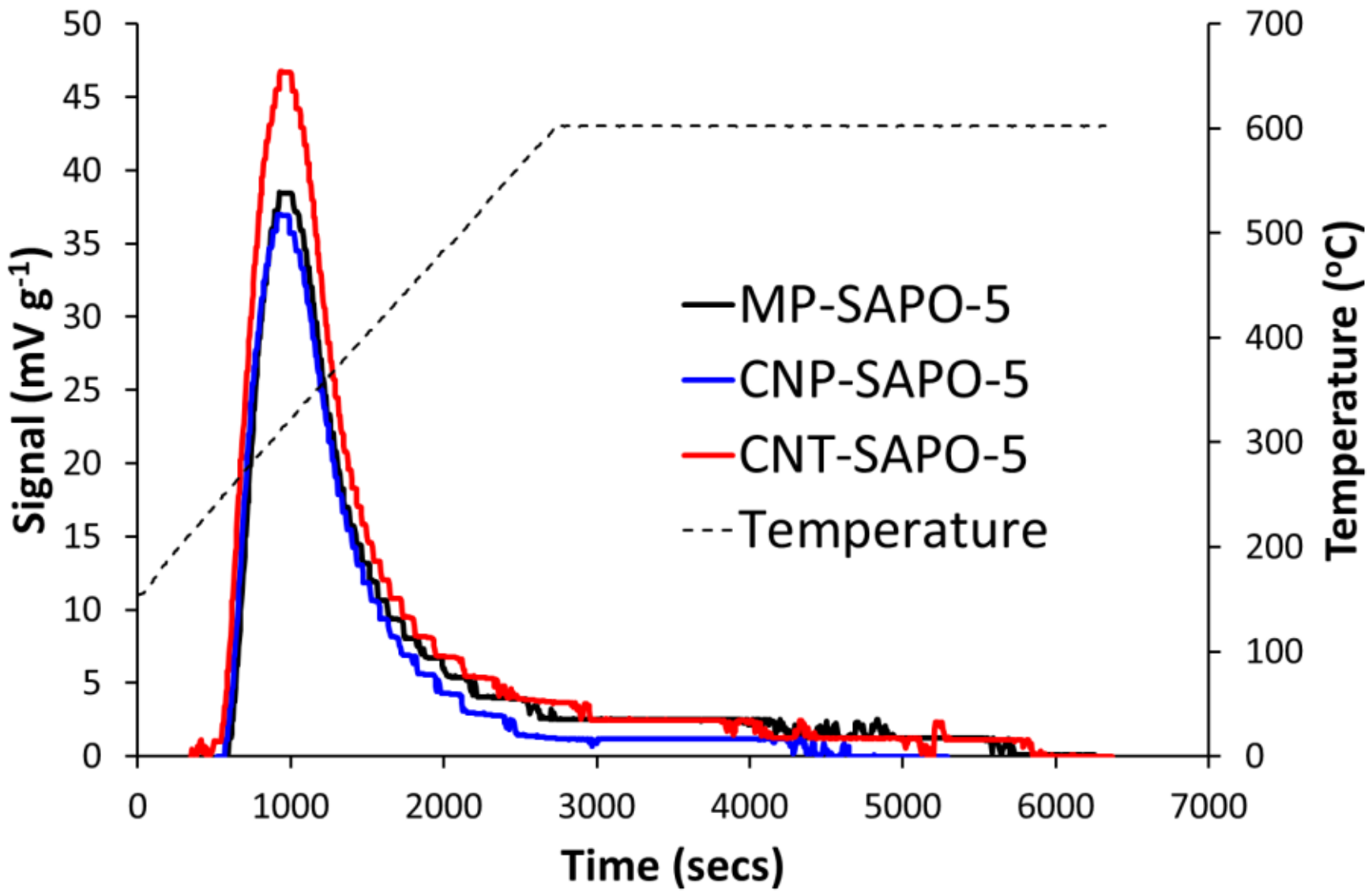

Fig. S2: NH_3-TPD profiles of the three SAPO-5 species, with the associated temperature ramp. Data is normalised per gram of sample for ease of comparison.

Table S3: Integrals and percentages of the three SAPO-5 systems, showing the influence of carbon templates on acidity.

**Sample**	**Integral (mV sec g^{-1})**								
	0 to 250 °C	250 to 300 °C	300 to 350 °C	350 to 400 °C	400 to 450 °C	450 to 500 °C	500 to 550 °C	550 to 600 °C	Total
**MP-SAPO-5**	4	6316	10643	5499	3014	1968	1329	6623	35396
**CNP-SAPO-5**	37	66341	10005	5412	2676	1509	802	2368	29450
**CNT-SAPO-5**	269	8621	13002	6838	3520	2322	1642	5839	42054

**Sample**	**Percentage (%)**								
	0 to 250 °C	250 to 300 °C	300 to 350 °C	350 to 400 °C	400 to 450 °C	450 to 500 °C	500 to 550 °C	550 to 600 °C	Total
**MP-SAPO-5**	0	18	30	16	9	6	4	19	100
**CNP-SAPO-5**	0	23	34	18	9	5	3	8	100
**CNT-SAPO-5**	1	21	31	16	8	6	4	14	100

Scanning electron microscopy (SEM) images

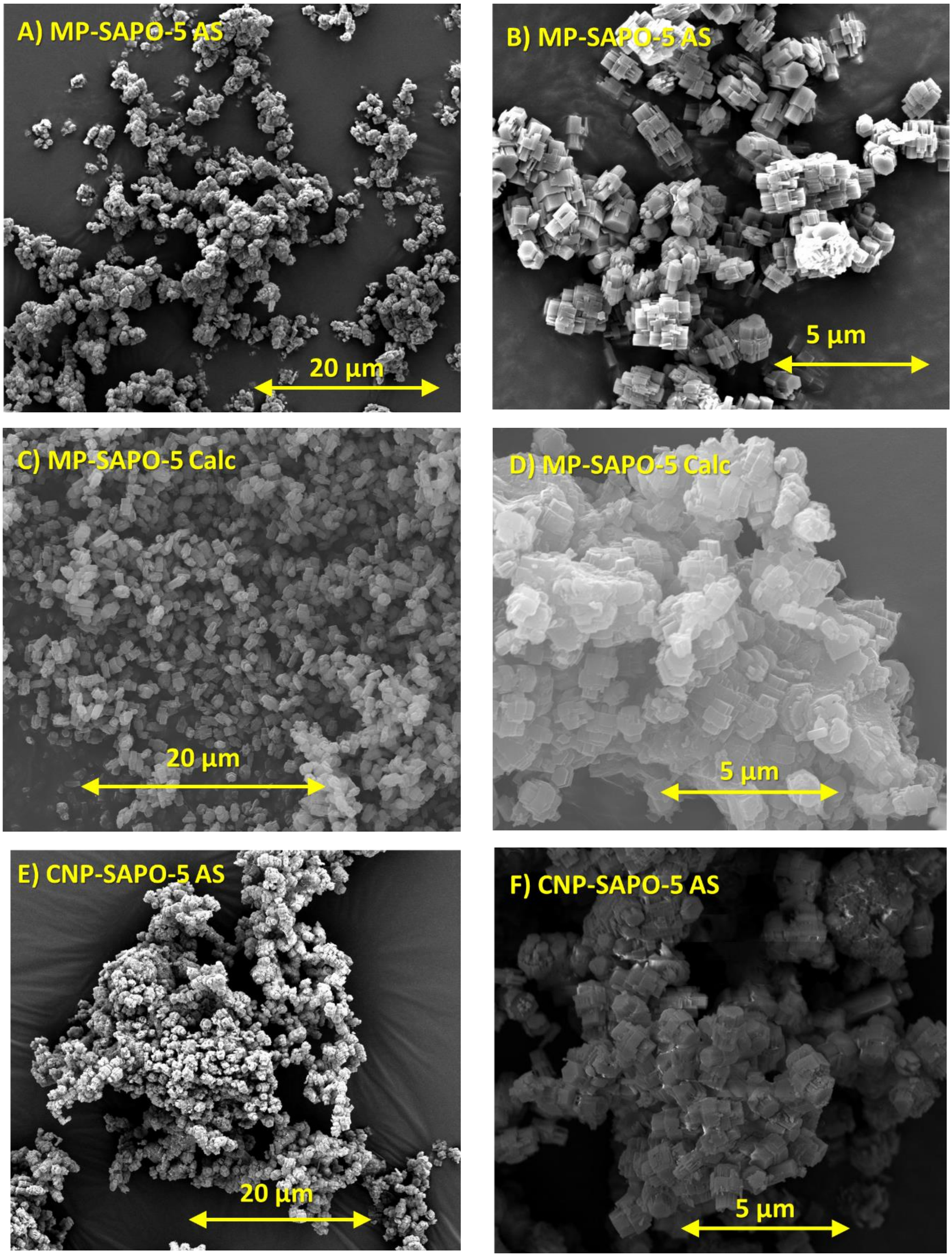

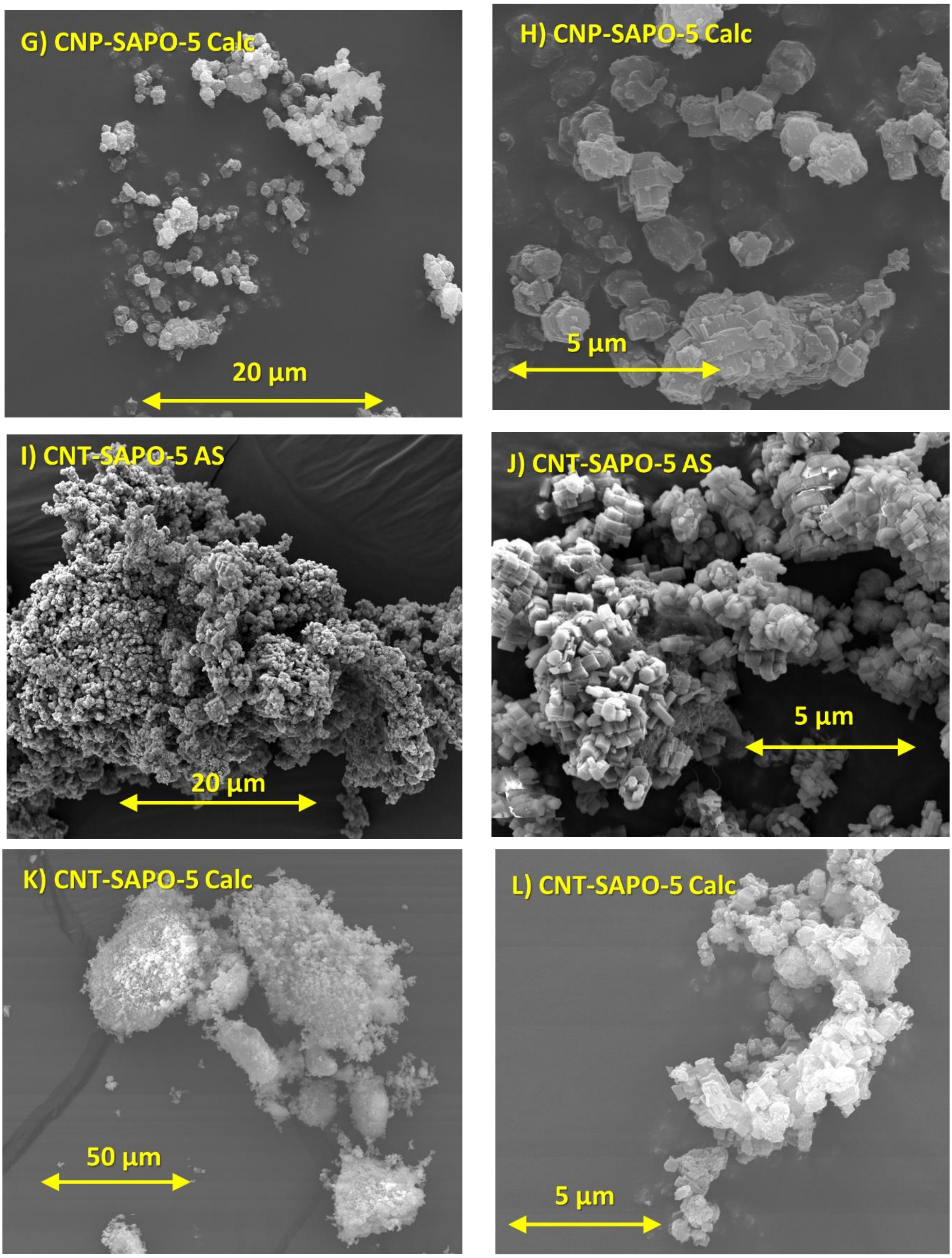

Fig. S3: Showing the particle size, shape and uniformity of the three SAPO-5 systems *via* SEM images, at different magnifications, focussing on A & B) MP-SAPO-5 AS, C & D) MP-SAPO-5 Calc, E & F) CNP-SAPO-5 AS, G & H) CNP-SAPO-5 Calc, I & J) CNT-SAPO-5 AS, and K & L) CNT-SAPO-5 Calc.

Transmission electron microscopy (TEM) images

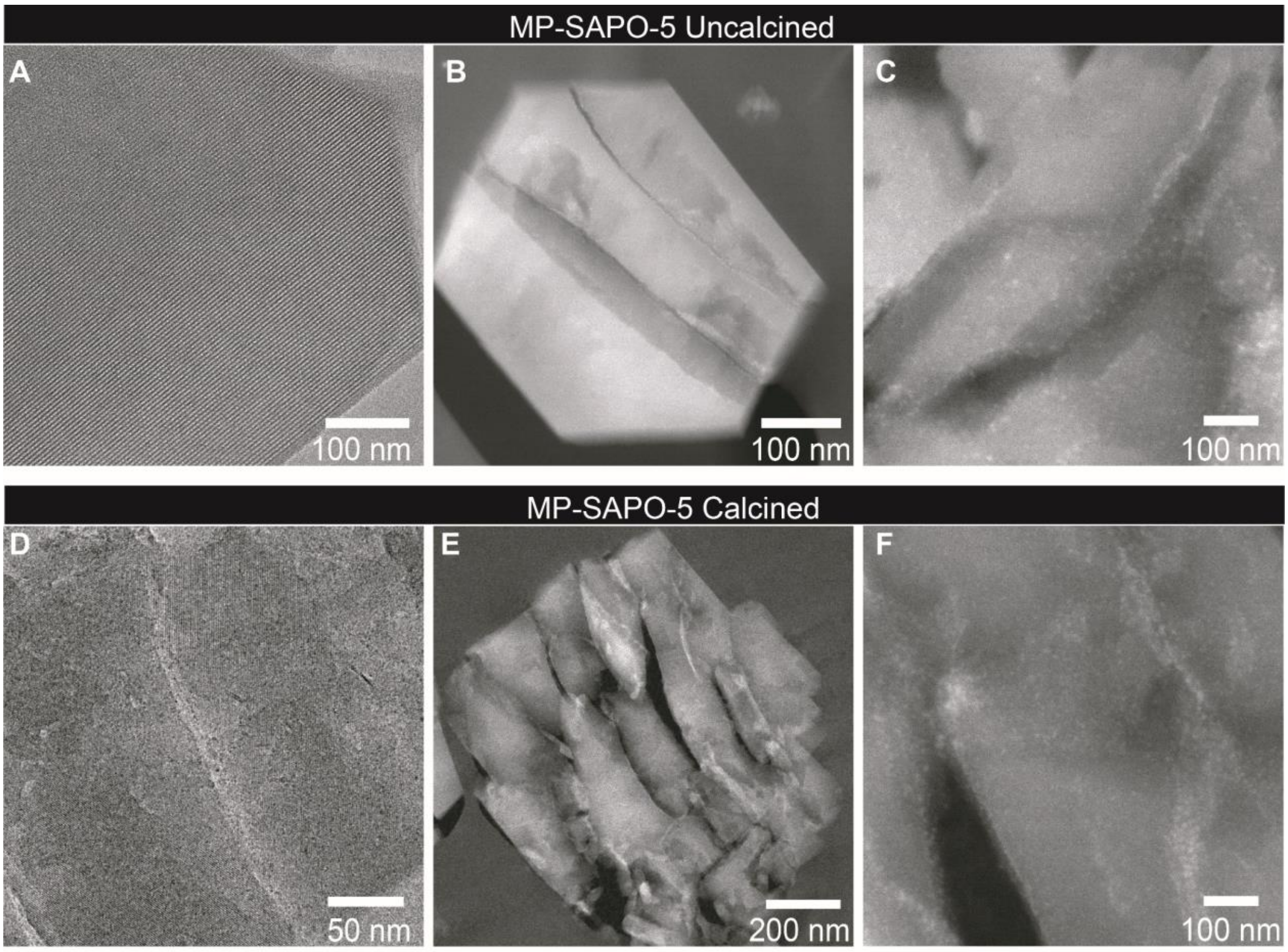

Fig. S4: Electron microscopy images of SAPO-5. A-C) Uncalcined and D-F) calcined. A,D) High resolution TEM images of the crystalline systems. B,E) Low resolution HAADF-STEM images show slight surface roughening after calcination. C,F) High resolution HAADF-STEM images show the presence of 1-2 nm nanoparticles.

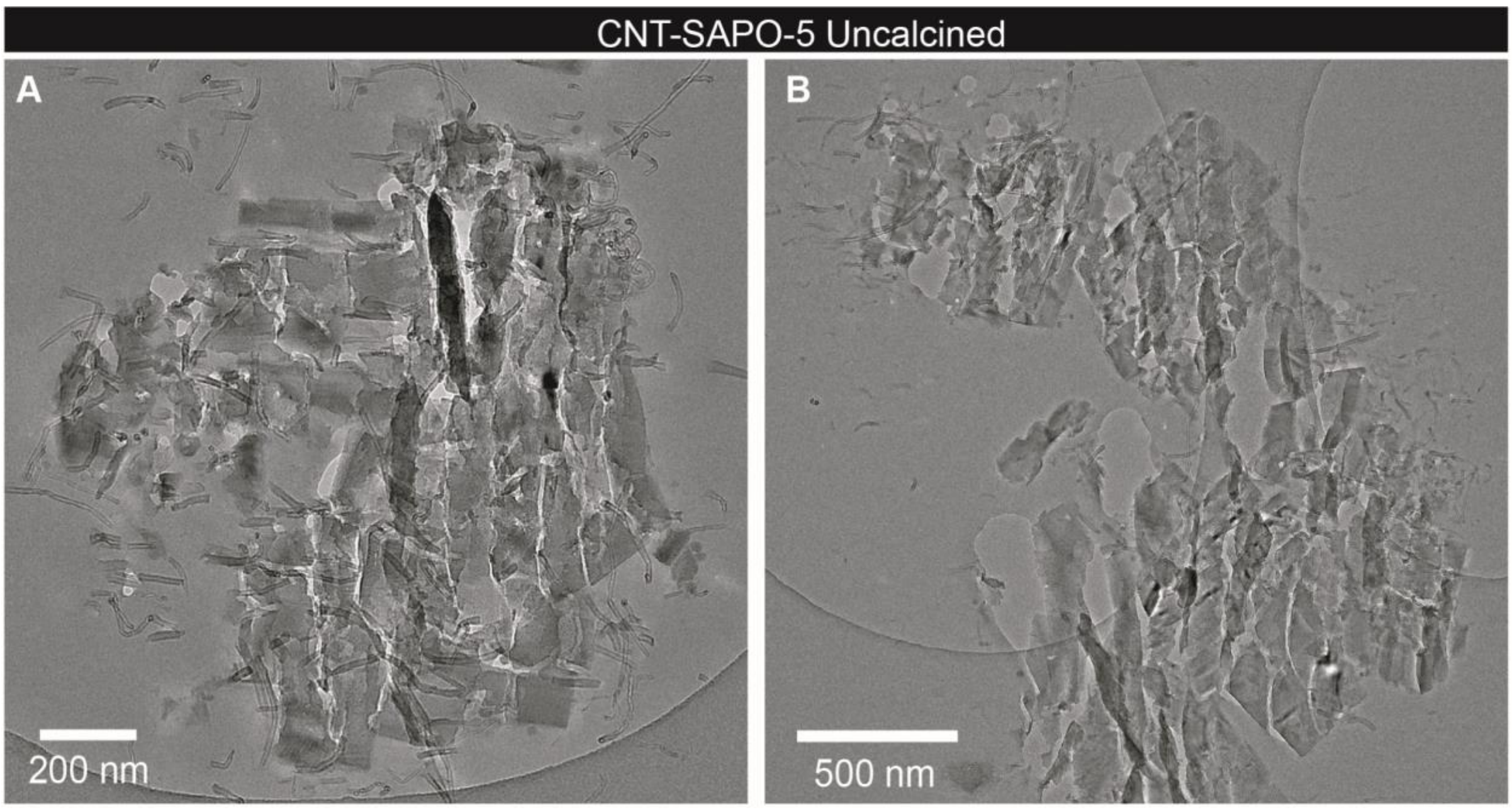

Fig. S5: Electron microscopy images of uncalcined CNT-SAPO-5 at different magnifications, showing the affinity between the SAPO-5 and CNT.

Comparison of MP-SAPO-5 Morphology

Whilst the same synthesis procedure was followed in this work as in our previous work[3], different batches of reagents were used, and this seems to have resulted in subtle morphological differences between the MP-SAPO-5 samples, see Figure S5. The crystals from the earlier work appear to be more discrete and better defined. There are consequently differences in the SANS between the two materials, most notable in the region $0.01 \leq Q \leq 0.1$ Å^{-1}. Whilst the two samples were collected on different instruments, they have been calibrated in an identical manner, so the difference in intensity between the samples (roughly a factor of 2) is likely due to the difference in packing between the two samples. Thus, the previous (more intense) data is likely packed more densely in the cells, and thus more is illuminated by the neutron beam, than in the current data.

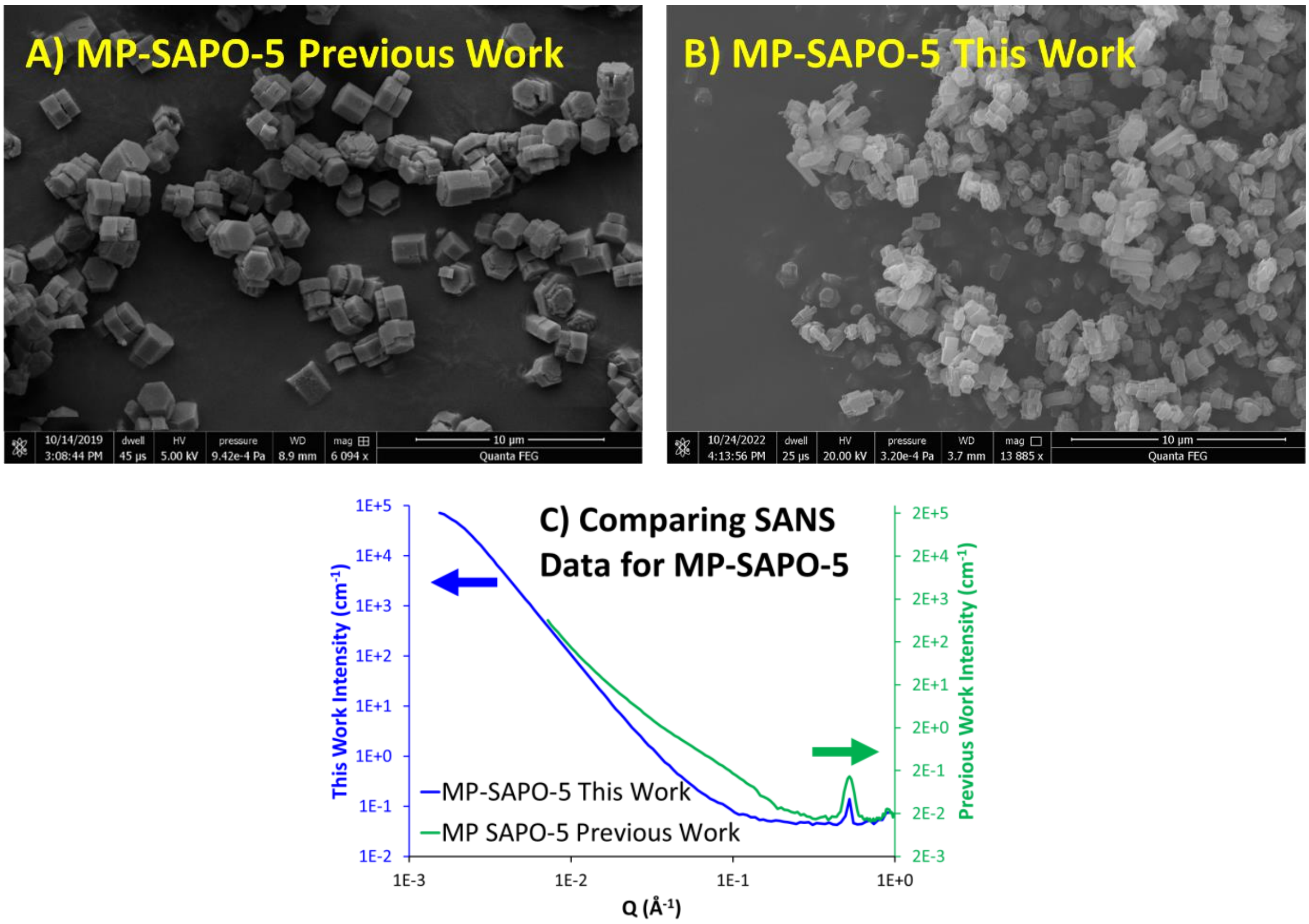

Fig. S6: SEM micrographs comparing the crystallinity of the MP-SAPO-5 species synthesized for A) our previous work described in reference 3 and B) this work, along with C) the corresponding SANS data (measured on the LOQ and SANS2D instruments at the ISIS Neutron and Muon Source, respectively).

Small angle neutron scattering (SANS) data fitting

Power law – Fractal – Gaussian Peak model

Table S4: Fitting parameters and values, with associated uncertainties, achieved with a model including a power law, fractal aggregate and Gaussian peak for the three SAPO-5 systems. Grey cells denote parameters that were not optimised.

**Parameter**	**MP SAPO-5**	**CNP-SAPO-5**	**CNT-SAPO-5**
Goodness of Fit			
Reduced χ^2	15.2	211.2	99.1
General Parameters			
**Background**, cm^{-1}	0.0377<br>± $7.9x10^{-4}$	0.0139<br>± $5.1x10^{-4}$	0.0360<br>± $3.5x10^{-4}$
Power Law			
Scale, **A**<br>(≥ 0)	$1.26x10^{-5}$<br>± $2.0x10^{-7}$	$4.54x10^{-8}$<br>± $5.9x10^{-8}$	$9.44x10^{-10}$<br>± $3.4x10^{-9}$
Exponent<br>(3 to 4)	3.00<br>± 0.01	3.04<br>± 0.09	3.18<br>± 0.28
Fractal Aggregate			
Scale, **B**<br>(≥ 0)	0.026<br>± $8.2x10^{-5}$	0.133<br>± $2.5x10^{-3}$	0.043<br>± $1.6x10^{-4}$
Radius, Å<br>(1 to 10)	4.42<br>± 0.13	1.25<br>± 0.13	1.00<br>± 0.03
Fractal Dimension<br>(2 to 3)	3.00<br>± 0.01	2.97<br>± 0.01	2.91<br>± 0.01
Correlation Length, Å	1205<br>± 5	11273<br>± 274	1326<br>± 7
SLD Pore, 10^{-6}, Å^{-2}<br>(Fixed)	0	0	0
SLD Matrix, 10^{-6}, Å^{-2}<br>(Fixed)	4.1	4.1	4.1
Radius Polydispersity[a]<br>(Fixed)	0.3	0.3	0.3
Gaussian Peak, (100) Bragg Peak			
Scale, **D**<br>(≥ 0)	32.87<br>± 5.1	1.70<br>± 0.31	93.46<br>± 20.7
Position, Å^{-1}<br>(0.48 to 0.58)	0.510<br>± $7.1x10^{-4}$	0.511<br>± $1.2x10^{-4}$	0.539<br>± $1.5x10^{-4}$
Width[b], Å^{-1}	0.0041<br>± $4.6x10^{-5}$	0.0055<br>± $1.7x10^{-5}$	0.0039<br>± $1.5x10^{-4}$

a) HWHM of Lognormal distribution at the median radius divided by the median radius.
b) One standard deviation = FWHM/2.354

This model is described by a linear combination of the following Q-dependent functions: a power law (to account for interfacial scattering), a fractal aggregate of polydisperse spheres (to account for micropores), and a Gaussian peak (to account for the Bragg peak):

$$\text{Intensity}(Q)_{P+F(PD)+G} = \text{Background} + A.f(Q)_{PowerLaw} + B.f(Q)_{Fractal(PD)} + D.f(Q)_{Gaussian}$$

Here, A, B & D are scalar quantities that are proportional to the volume fraction of that component in the model. Links to the individual model descriptions may be found below.

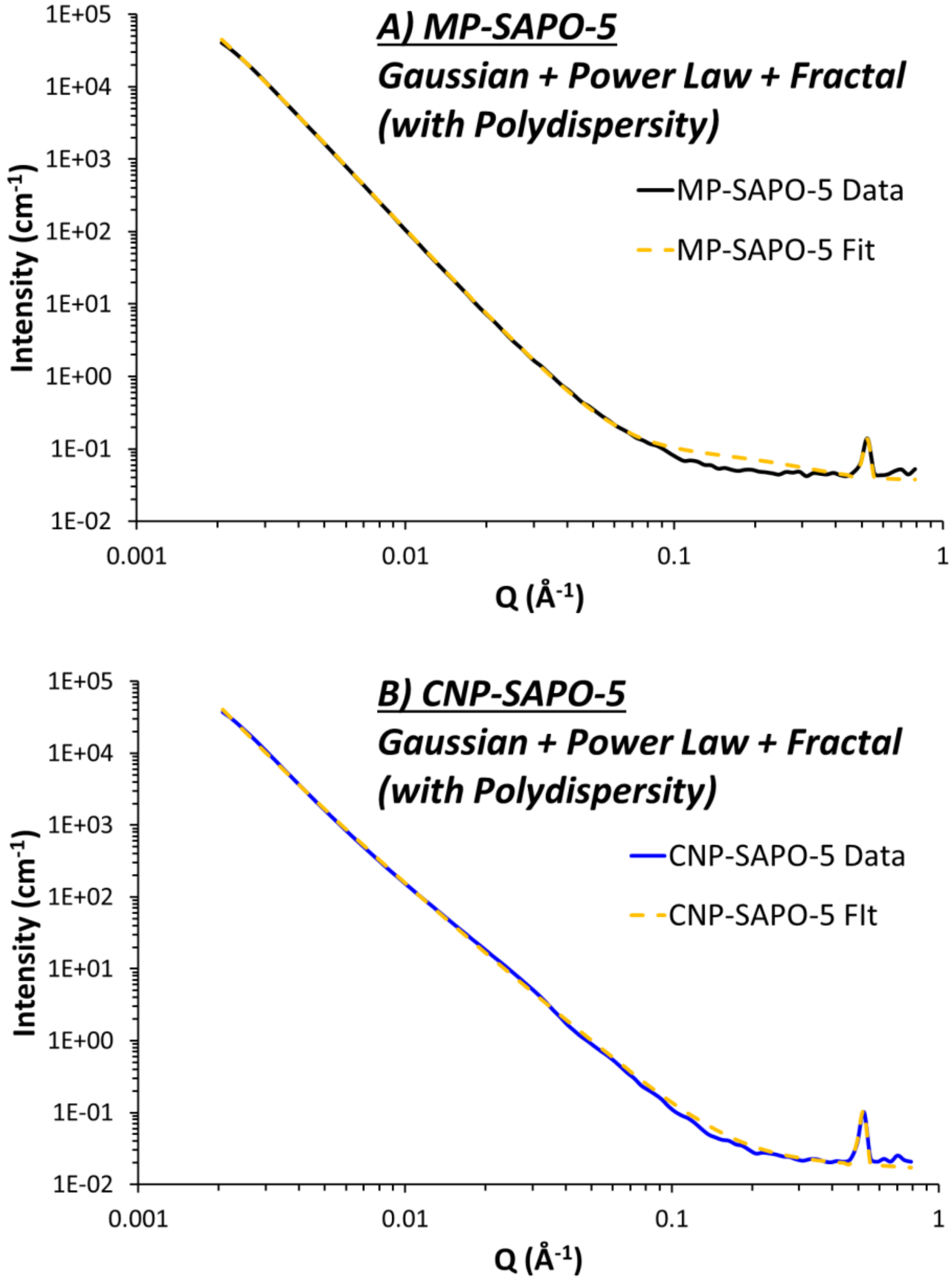

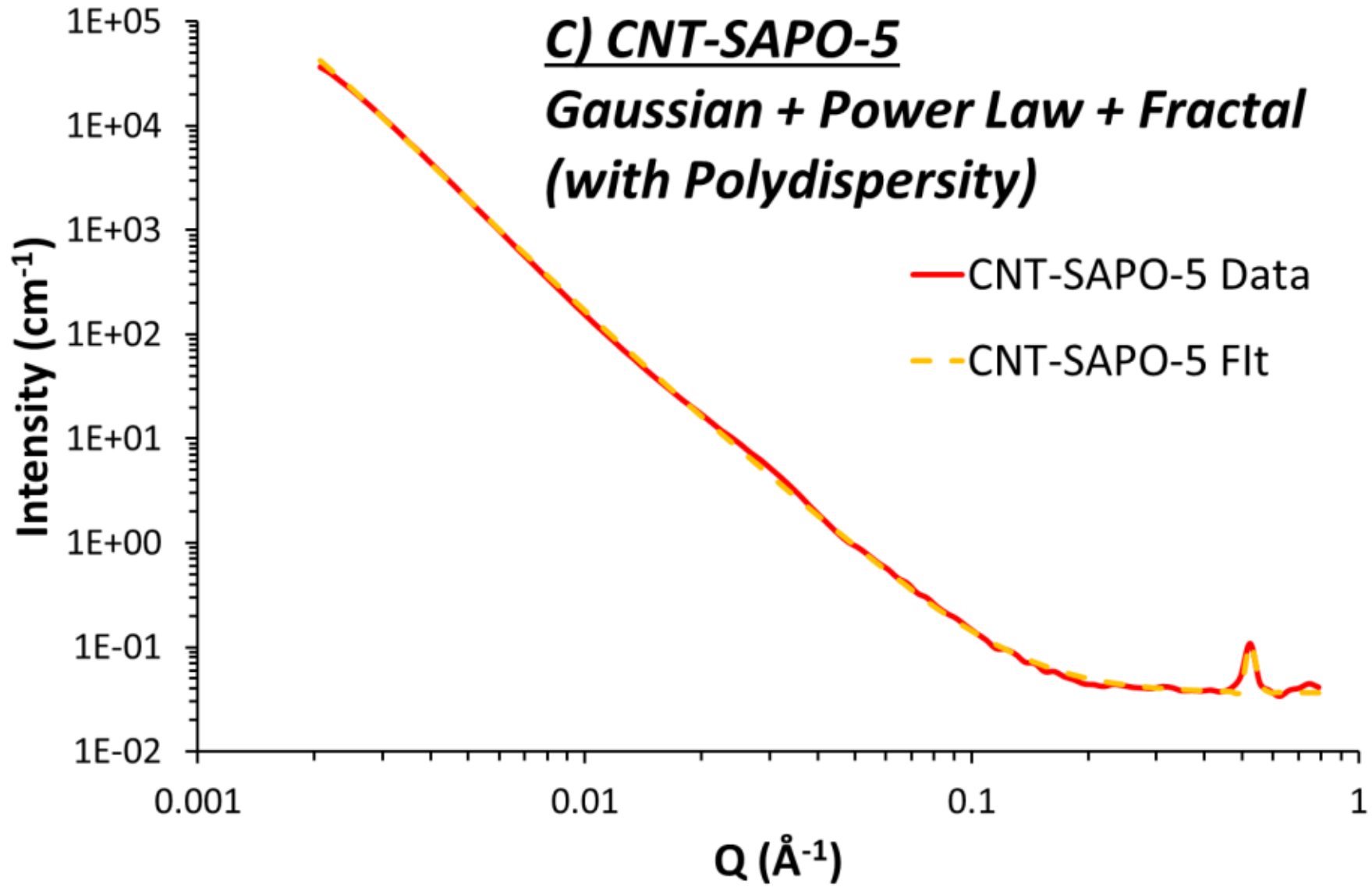

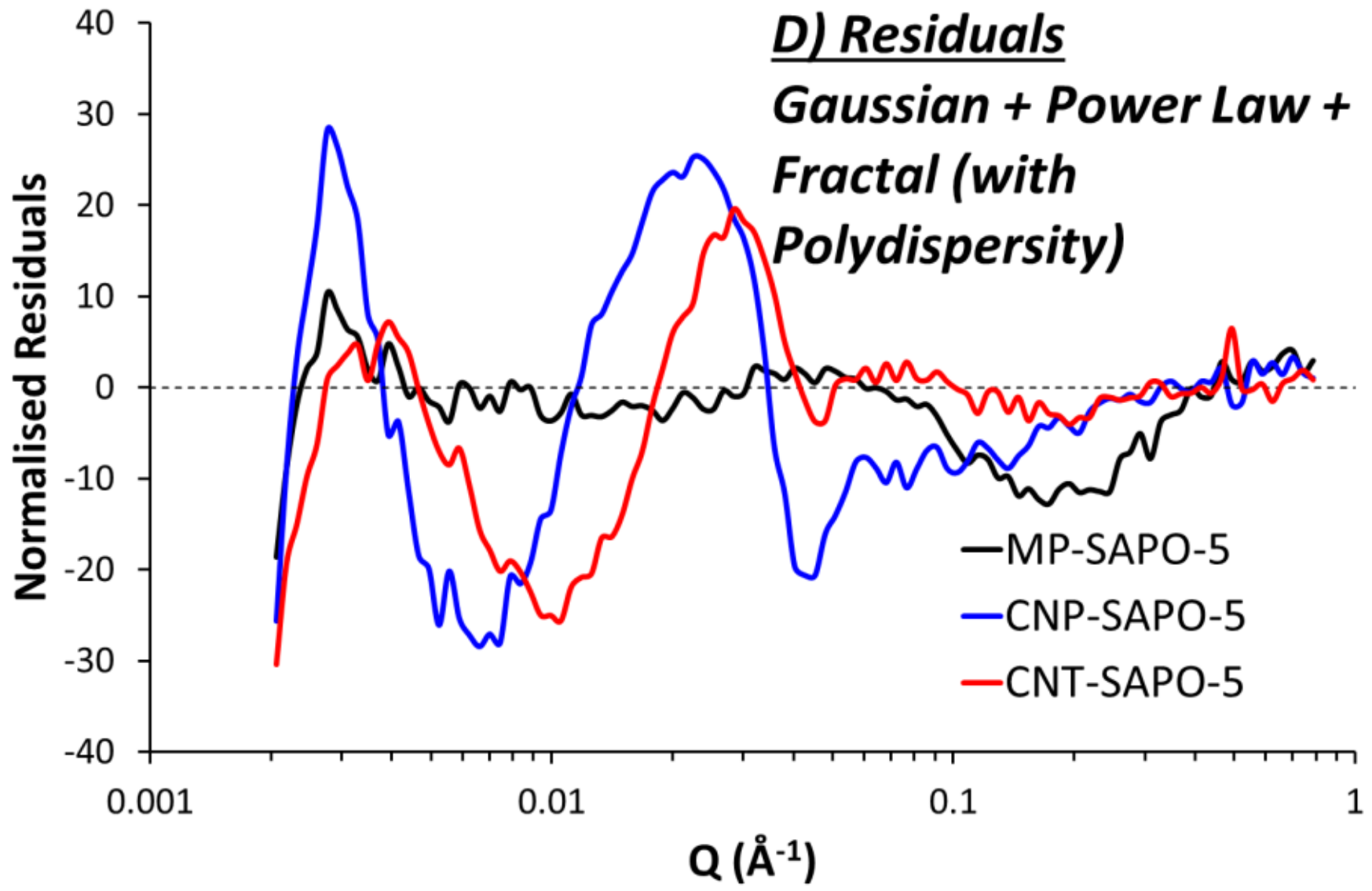

Fig. S7: (A-C) Fits of the Power law – Fractal - Gaussian model as defined above to the SANS data from MP-SAPO-5, CNP-SAPO-5 and CNT-SAPO-5, respectively. (D) Residual intensity (observed – calculated) from these fits, serving as a measure of fit quality.

Power law – Fractal – Monodisperse Sphere – Gaussian Peak Model

Table S5: Fitting parameters and values, with associated uncertainties, achieved with a model including a power law, fractal aggregate, sphere and Gaussian peak for the three SAPO-5 systems. Grey cells denote parameters that were not optimised.

Parameter	MP SAPO-5	CNP-SAPO-5	CNT-SAPO-5
Goodness of fit			
Reduced χ^2	8.4	48.4	35.0
General parameters			
**Background**, cm^{-1}	0.0431	0.0171	0.0301

	± 9.2x10^{-4}	± 4.0x10^{-4}	± 1.0x10^{-3}
Power Law			
Scale, **A** (≥ 0)	8.96x10^{-6} ± 3.4x10^{-7}	3.47x10^{-8} ± 7.1x10^{-8}	7.94x10^{-9} ± 1.1x10^{-7}
Exponent (3 to 4)	3.00 ± 0.01	3.12 ± 0.13	3.10 ± 0.12
Fractal Aggregate			
Scale, **B** (≥ 0)	0.026 ± 8.2x10^{-5}	0.059 ± 8.9x10^{-4}	0.032 ± 2.0x10^{-4}
Radius, Å (1 to 10)	1.89 ± 0.23	1.43 ± 0.16	2.88 ± 0.14
Fractal Dimension (2 to 3)	3.00 ± 0.01	2.94 ± 0.01	2.94 ± 0.01
Correlation Length, Å	1159 ± 5	3249 ± 73	1008 ± 6
SLD Pore, 10^{-6}, Å^{-2} (Fixed)	0	0	0
SLD Matrix, 10^{-6}, Å^{-2} (Fixed)	4.1	4.1	4.1
Radius Polydispersity[a] (Fixed)	0.3	0.3	0.3
Sphere			
Scale, **C** (≥ 0)	0.00069 ± 3.4x10^{-5}	0.00129 ± 1.1x10^{-5}	0.00180 ± 2.7x10^{-5}
SLD Pore, 10^{-6}, Å^{-2} (Fixed)	0	0	0
SLD Matrix, 10^{-6}, Å^{-2} (Fixed)	4.1	4.1	4.1
Radius, Å	30 ± 1	102 ± 1	74 ± 1
Radius Polydispersity (Fixed)	0	0	0
Gaussian Peak, (100) Bragg Peak			
Scale, **D** (≥ 0)	3.73 ± 0.6	9.76 ± 1.9	18.08 ± 7.7
Position, Å^{-1} (0.48 to 0.58)	0.511 ± 9.9x10^{-5}	0.510 ± 6.4x10^{-5}	0.510 ± 1.0x10^{-4}
Width[b], Å^{-1}	0.0050 ± 8.3x10^{-5}	0.0045 ± 8.5x10^{-5}	0.0042 ± 9.3x10^{-5}

a) HWHM of Lognormal distribution at the median radius divided by the median radius.
b) One standard deviation = FWHM/2.354

This model is described by a linear combination of the following Q-dependent functions: a power law (to account for interfacial scattering), a fractal aggegrate of polydisperse spheres (to account for micropores), monodisperse spheres (to account for mesopores), and a Gaussian peak (to account for the Bragg peak):

$$\text{Intensity}(Q)_{P+F(PD)+S+G} = \text{Background} + A.f(Q)_{PowerLaw} + B.f(Q)_{Fractal(PD)} + C.f(Q)_{Sphere} + D.f(Q)_{Gaussian}$$

Here, A, B, C & D are scalar quantities that are proportional to the volume fraction of that component in the model. Links to the individual model descriptions may be found below.

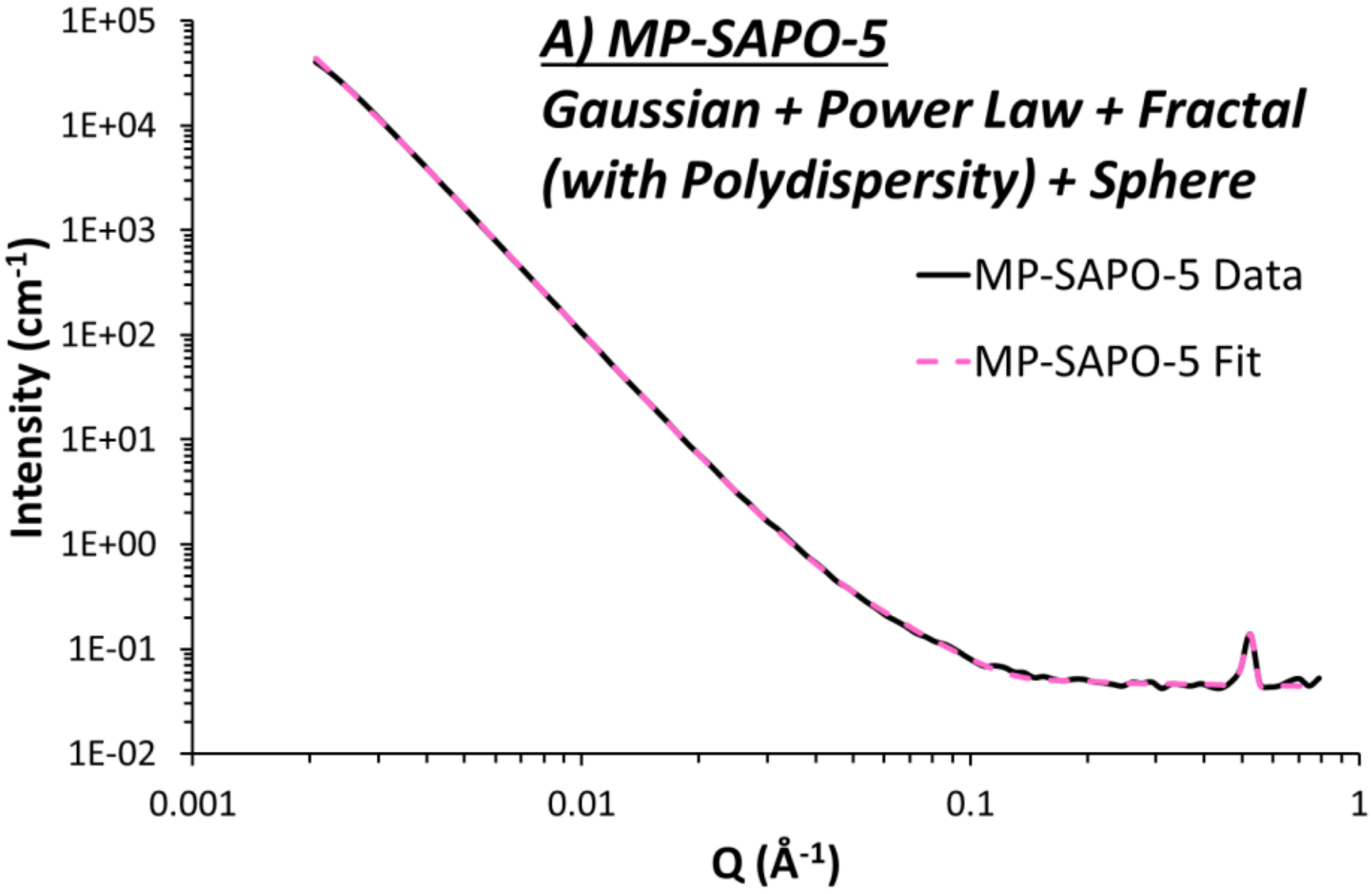

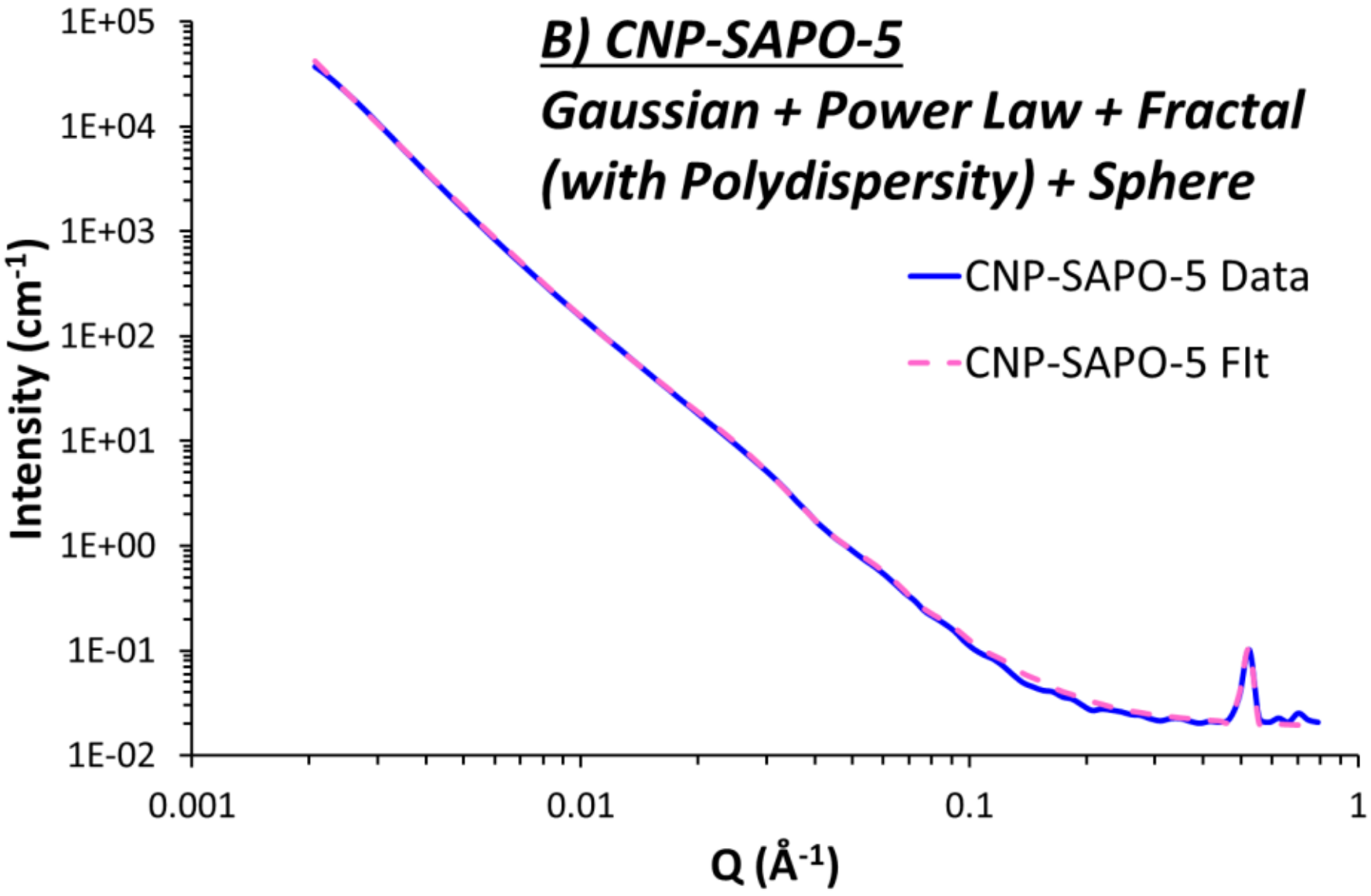

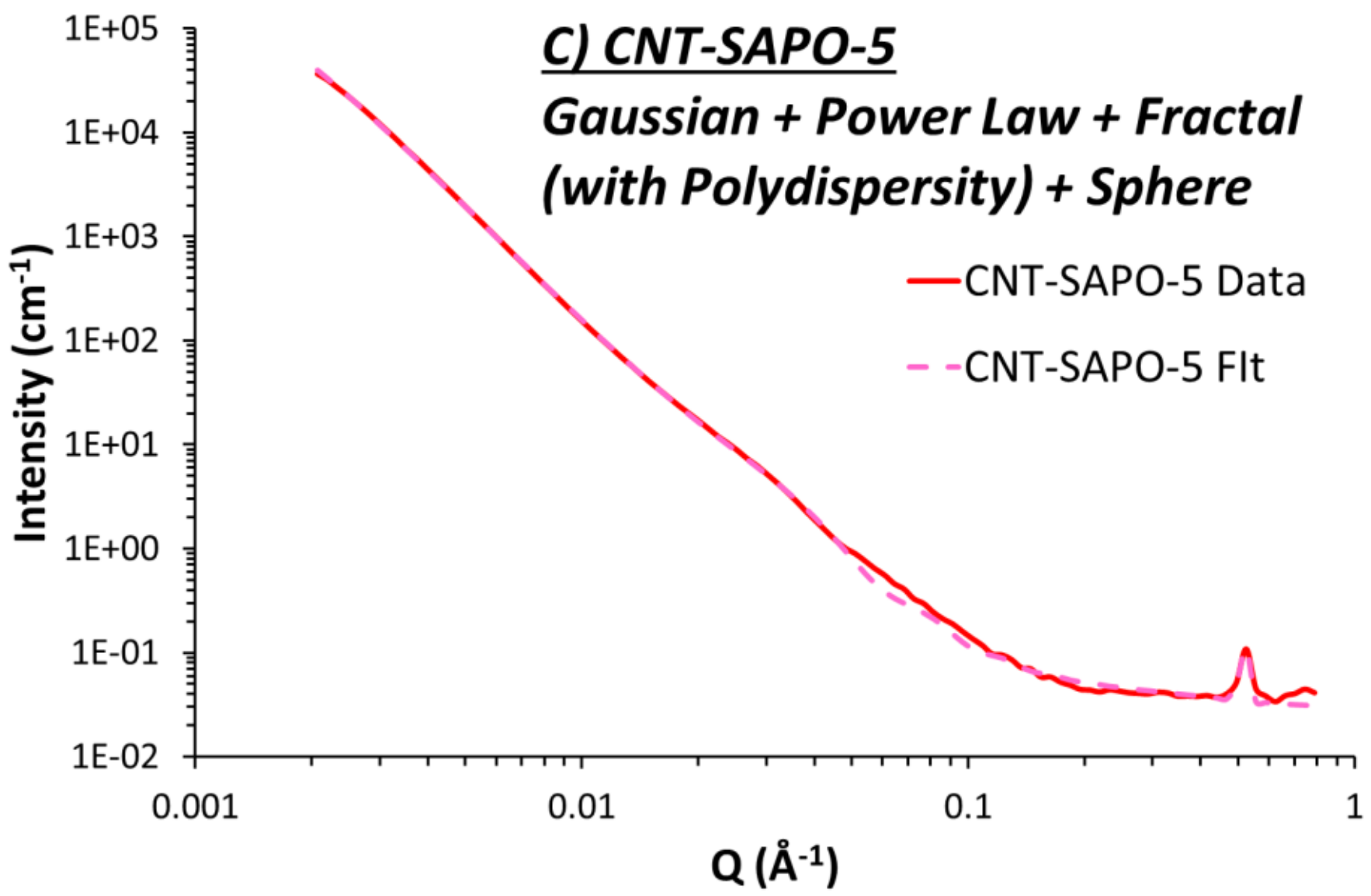

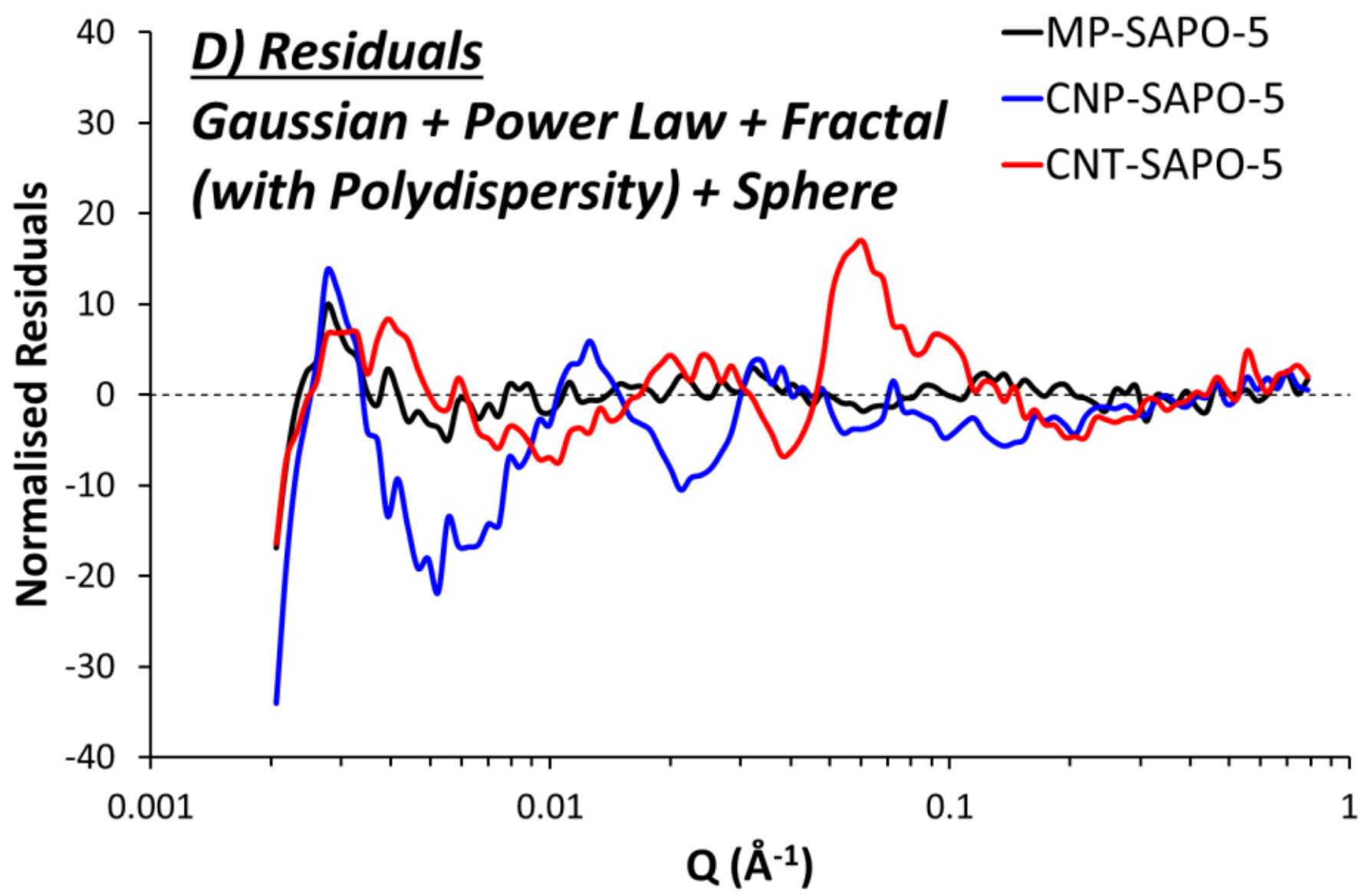

Fig. S8: (A-C) Fits of the Power law – Fractal – Sphere - Gaussian model as defined above to the SANS data from MP-SAPO-5, CNP-SAPO-5 and CNT-SAPO-5, respectively. (D) Residual intensity (observed – calculated) from these fits, serving as a measure of fit quality.

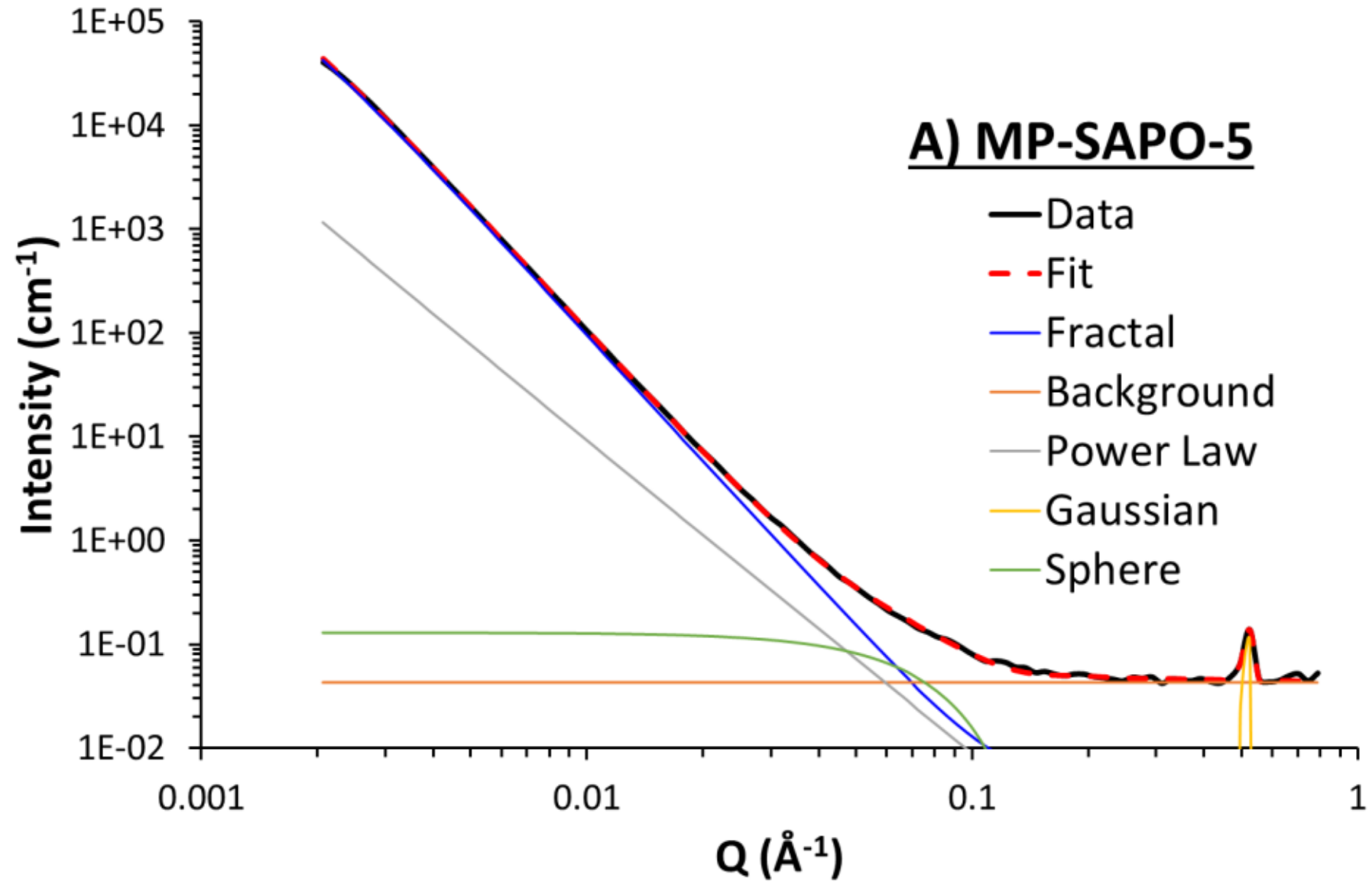
A) MP-SAPO-5
Data
Fit
Fractal
Background
Power Law
Gaussian
Sphere
Intensity (cm-1)
Q (Å-1)
1E+05
1E+04
1E+03
1E+02
1E+01
1E+00
1E-01
1E-02
0.001
0.01
0.1
1

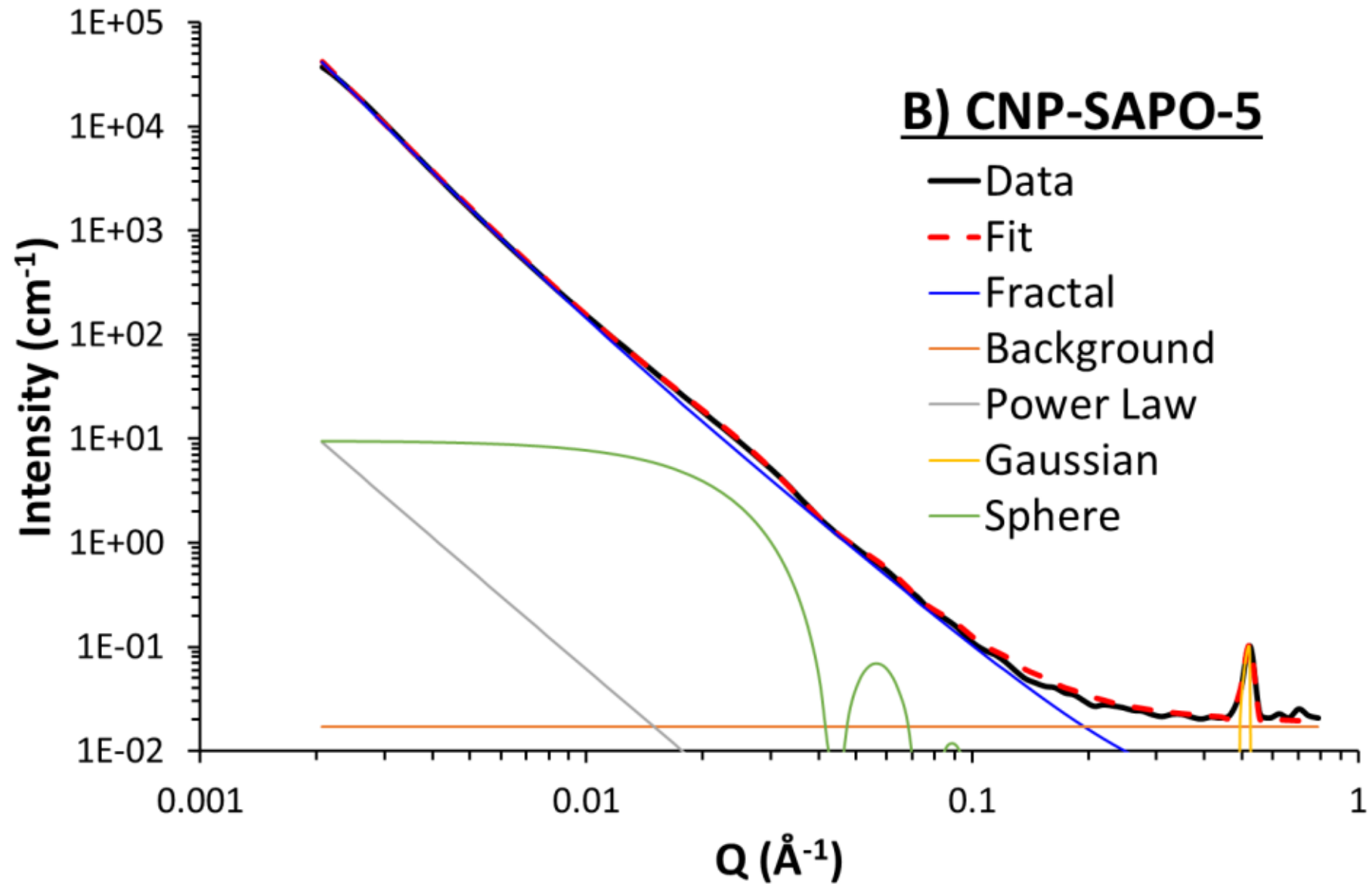
B) CNP-SAPO-5
Data
Fit
Fractal
Background
Power Law
Gaussian
Sphere
Intensity (cm-1)
Q (Å-1)
1E+05
1E+04
1E+03
1E+02
1E+01
1E+00
1E-01
1E-02
0.001
0.01
0.1
1

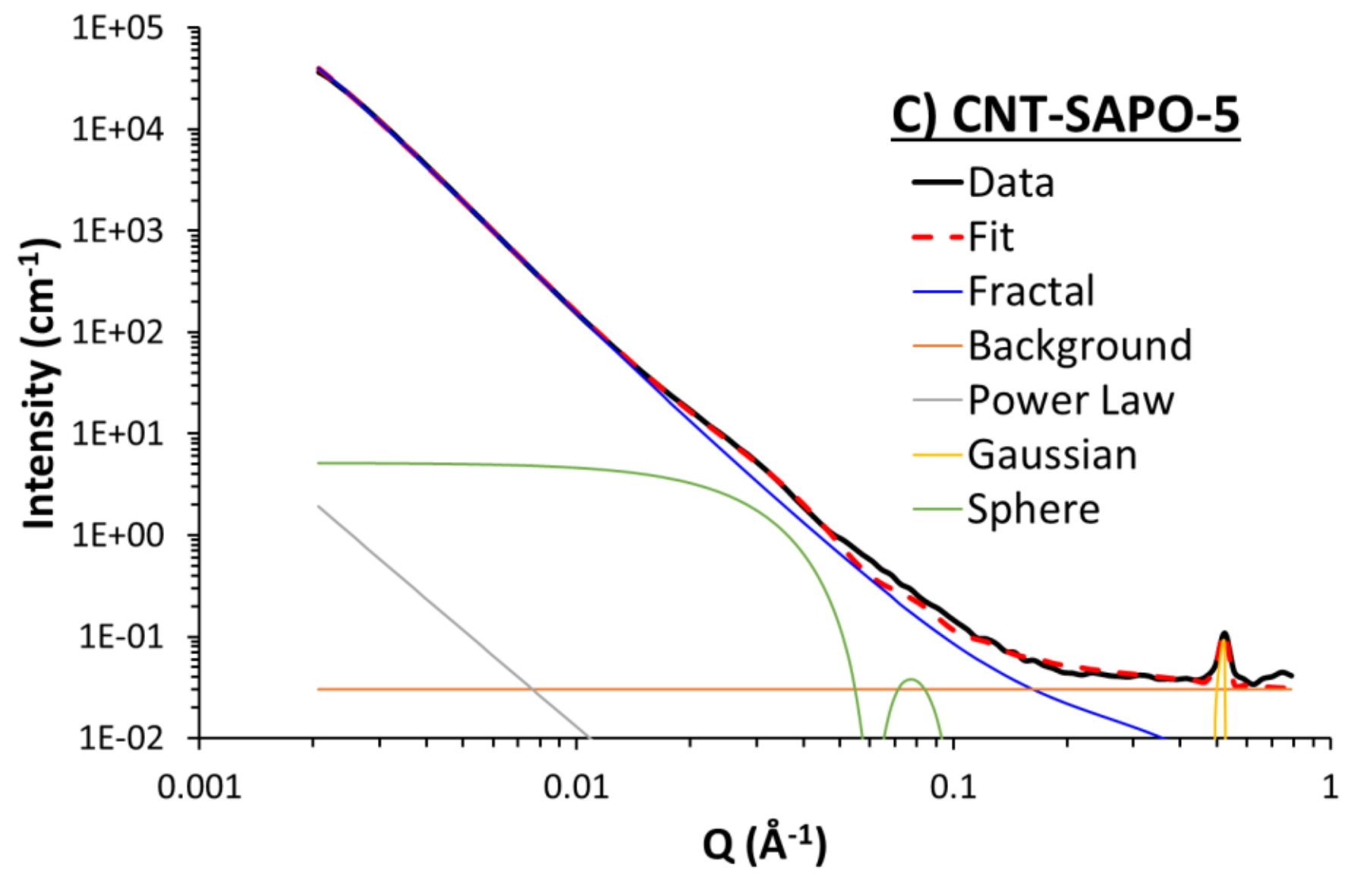
C) CNT-SAPO-5
Data
Fit
Fractal
Background
Power Law
Gaussian
Sphere
Intensity (cm-1)
Q (Å-1)
1E+05
1E+04
1E+03
1E+02
1E+01
1E+00
1E-01
1E-02
0.001
0.01
0.1
1

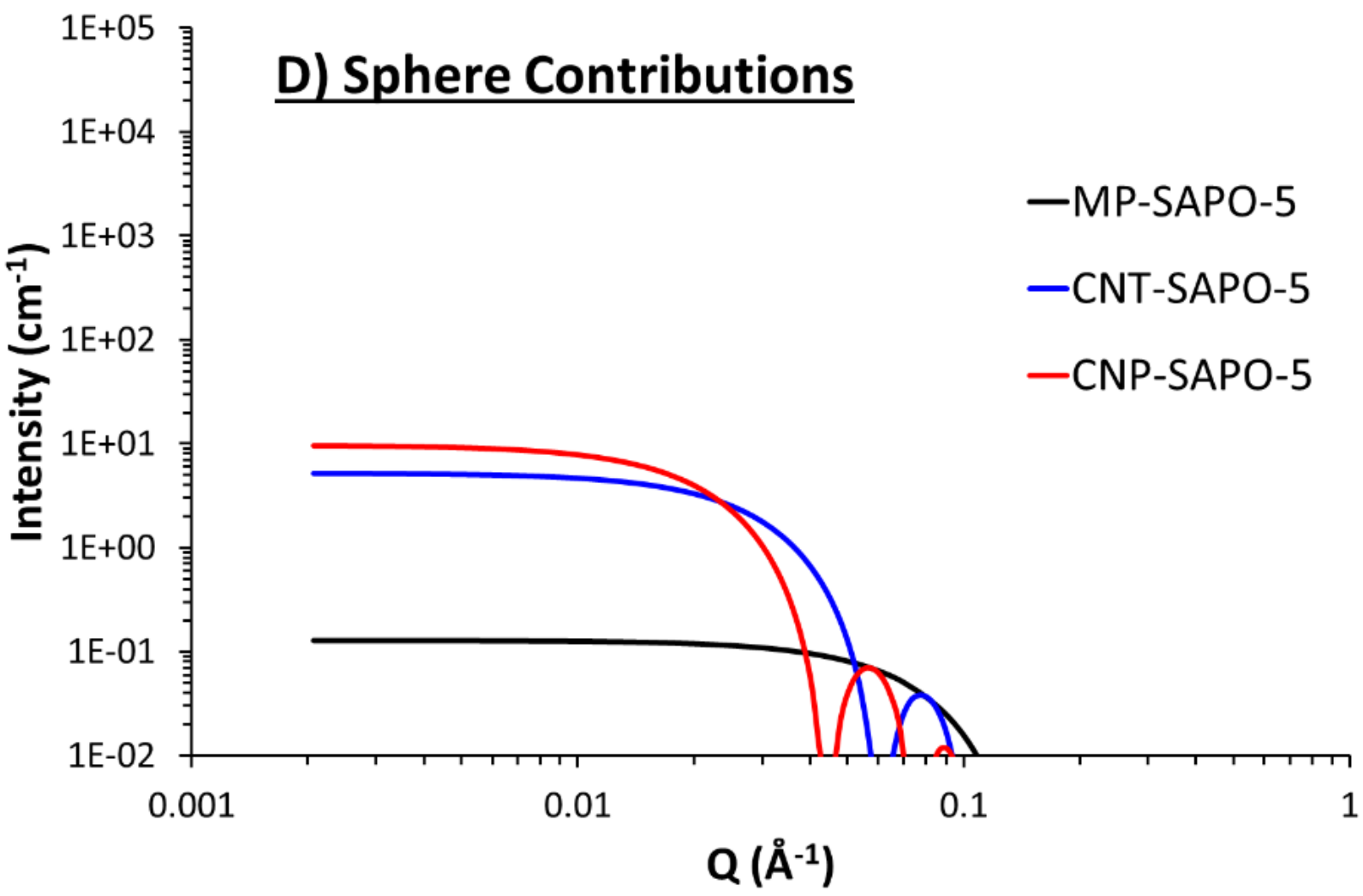

Fig. S9: Fits of the Power law – Fractal – Sphere - Gaussian model as defined above to the SANS data from MP-SAPO-5, CNP-SAPO-5 and CNT-SAPO-5, respectively. (A-C) Showing the contributions of the individual component functions. (D) Comparing just the Sphere contributions.

Power law – Fractal – Polydisperse Sphere – Gaussian Peak Model

Table S6: Fitting parameters and values, with associated uncertainties, achieved with a model including a power law, fractal aggregate, sphere (with variable polydispersity) and Gaussian peak for the three SAPO-5 systems. Grey cells denote parameters that were not optimised.

**Parameter**	**MP SAPO-5**	**CNP-SAPO-5**	**CNT-SAPO-5**
Goodness of fit			
Reduced χ^2	8.0	39.1	11.8
General parameters			
**Background**, cm^{-1}	0.0452 ± 6.9x10^{-4}	0.0178 ± 4.6x10^{-4}	0.0367 ± 3.3x10^{-3}
Power Law			
Scale, **A** (≥ 0)	7.89x10^{-6} ± 4.8x10^{-7}	1.24x10^{-8} ± 5.5x10^{-9}	4.46x10^{-7} ± 2.6x10^{-7}
Exponent (3 to 4)	3.00 ± 0.01	3.65 ± 0.09	3.13 ± 0.13
Fractal Aggregate			
Scale, **B** (≥ 0)	0.026 ± 1.0x10^{-4}	0.059 ± 8.0x10^{-4}	0.028 ± 5.2x10^{-4}
Radius, Å (1 to 10)	1.11 ± 0.22	1.04 ± 0.08	3.03 ± 0.68
Fractal Dimension (2 to 3)	3.00 ± 0.01	2.95 ± 0.01	2.96 ± 0.01
Correlation Length, Å	1149 ± 5	3255 ± 29	879 ± 17
SLD Pore, 10^{-6}, Å^{-2}	0	0	0

(Fixed)			
SLD Matrix, 10^{-6}, Å^{-2} (Fixed)	4.1	4.1	4.1
Radius Polydispersity[a] (Fixed)	0.3	0.3	0.3
Sphere			
Scale, **C** (≥ 0)	0.00100 ± 6.4×10^{-5}	0.00174 ± 5.1×10^{-5}	0.00395 ± 1.6×10^{-4}
SLD Pore, 10^{-6}, Å^{-2} (Fixed)	0	0	0
SLD Matrix, 10^{-6}, Å^{-2} (Fixed)	4.1	4.1	4.1
Radius, Å	12 ± 2	80 ± 2	18 ± 3
**Radius Polydispersity[a] (0 to 1)**	**0.47**	**0.28**	**0.69**
Gaussian Peak, (100) Bragg Peak			
Scale, **D** (≥ 0)	13.87 ± 3.1	9.83 ± 2.6	0.21 ± 0.1
Position, Å^{-1} (0.48 to 0.58)	0.510 ± 8.8×10^{-5}	0.511 ± 1.0×10^{-4}	0.513 ± 4.2×10^{-3}
Width[b], Å^{-1}	0.0043 ± 8.8×10^{-5}	0.0044 ± 8.9×10^{-5}	0.0074 ± 2.2×10^{-3}

a) HWHM of Lognormal distribution at the median radius divided by the median radius.
b) One standard deviation = FWHM/2.354

This model is described by a linear combination of the following Q-dependent functions: a power law (to account for interfacial scattering), a fractal aggregate of polydisperse spheres (to account for micropores), polydisperse spheres (to account for mesopores), and a Gaussian peak (to account for the Bragg peak):

$$\text{Intensity}(Q)_{P+F(PD)+S+G} = \text{Background} + A.f(Q)_{PowerLaw} + B.f(Q)_{Fractal(PD)} + C.f(Q)_{Sphere} + D.f(Q)_{Gaussian}$$

Here, A, B, C & D are scalar quantities that are proportional to the volume fraction of that component in the model. Links to the individual model descriptions may be found below.

In this last set of model fits the uniformity of the mesopore sizes in the three samples was explored by including polydispersity on the spherical form factor. As can be seen, this had little influence on the parameters for the MP-SAPO-5 system, where χ^2 improved slightly from 8.4 to 8.0. For CNP-SAPO-5, χ^2 improved from 48.4 to 39.1. However, for CNT-SAPO-5 the fit improved significantly, with χ^2 falling from 35.0 to 11.8. This reinforces the theory that the mesopores in the CNT-SAPO-5 species are less uniform than the CNP-SAPO-5 species.

Descriptions of model components

Power Law: http://www.sasview.org/docs/user/models/power_law.html
Fractal: http://www.sasview.org/docs/user/models/fractal.html
Sphere: http://www.sasview.org/docs/user/models/sphere.html
Gaussian Peak: http://www.sasview.org/docs/user/models/gaussian_peak.html

__**Catalytic ethanol dehydration data**__

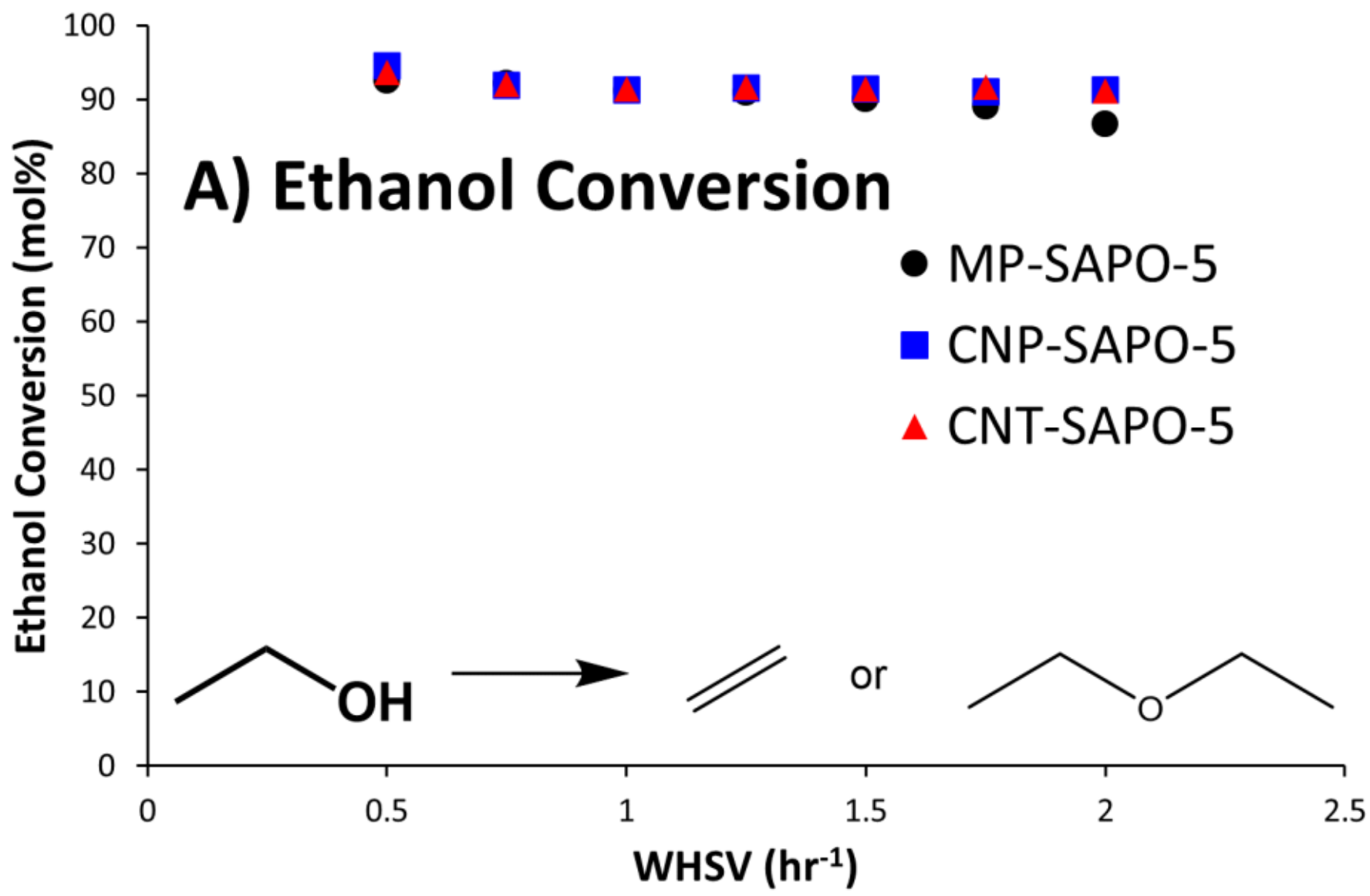

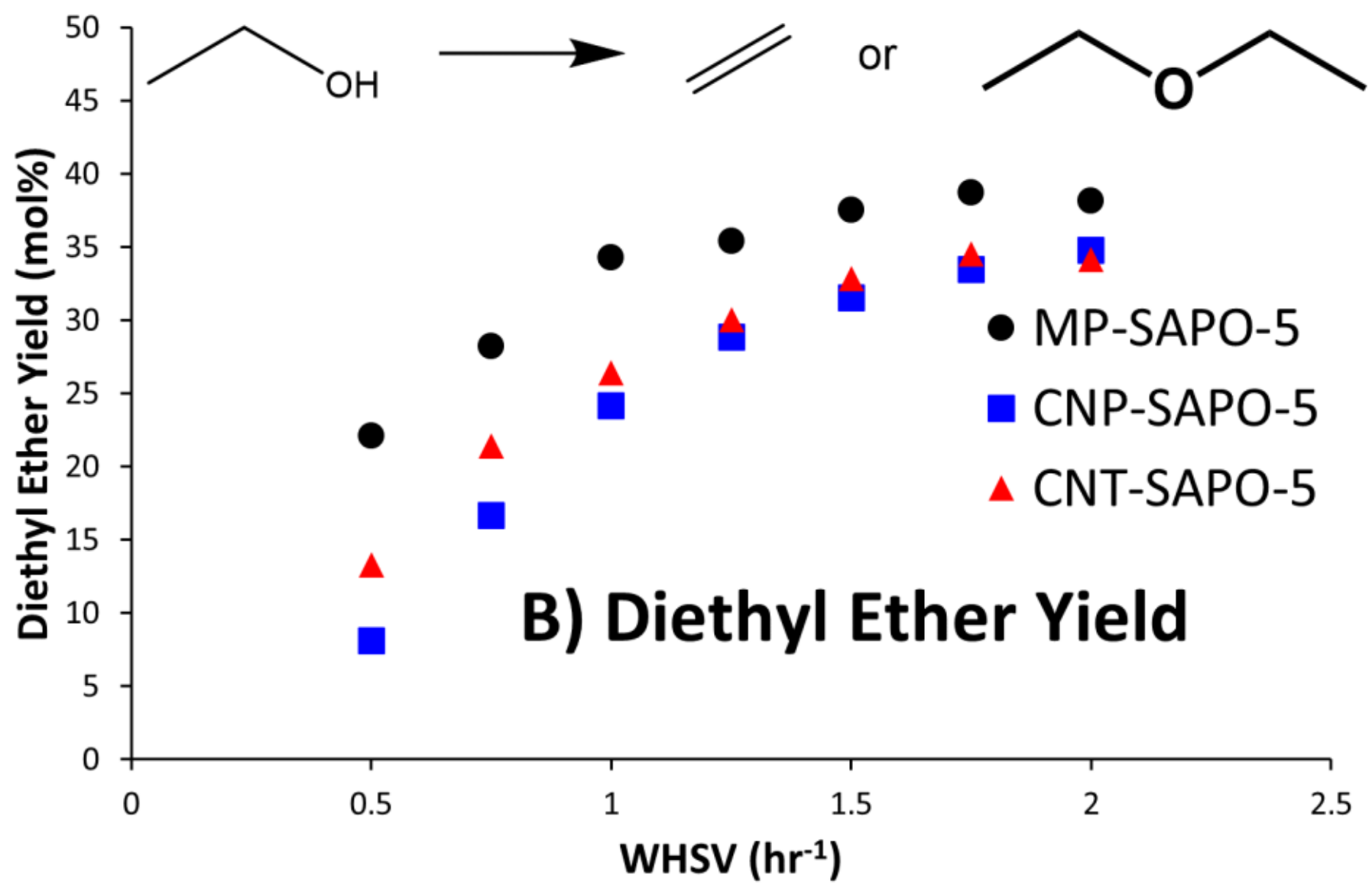

Fig. S10: Catalytic data for ethanol dehydration with the three SAPO-5 systems showing A) ethanol conversion and B) diethyl ether yield. Conditions: 230 °C, 25 mL/min N_2 carrier gas, liquid feedstock of 10% heptane (internal standard) in ethanol, liquid flow varied from 3.13 to 12.51 μL min^{-1} as the WHSV varies from 0.5 to 2.0 hr^{-1}. Errors are not shown but are calculated as ± 3 mol% based on multiple injections.

Table S7: Numerical catalytic data for ethanol dehydration with the three SAPO-5 systems showing Conditions: 230 °C, 25 mL/min N_2 carrier gas, liquid feedstock of 10% heptane (internal standard) in ethanol, liquid flow varied from 3.13 to 12.51 µL min^{-1} as the WHSV varies from 0.5 to 2.0 hr^{-1}. Errors are not shown but calculated as ± 3 mol% based on multiple injections.

		WHSV (hr^{-1})						
**Sample**	**Parameter (mol%)**	**0.5**	**0.75**	**1.0**	**1.25**	**1.5**	**1.75**	**2.0**
**MP-SAPO-5**	Ethanol Conversion	92.5	92.2	91.2	90.9	90.0	89.1	86.6
	Ethylene Yield	46.7	34.5	21.2	18.4	13.8	10.8	9.3
	Diethyl Ether Yield	22.1	28.2	34.3	35.4	37.5	38.7	38.2
**CNP-SAPO-5**	Ethanol Conversion	94.5	91.9	91.3	91.6	91.4	91.1	91.2
	Ethylene Yield	78.2	58.4	42.6	33.4	28.0	23.8	21.3
	Diethyl Ether Yield	8.1	16.7	24.2	28.9	31.5	33.5	34.8
**CNT-SAPO-5**	Ethanol Conversion	93.7	92.0	91.4	91.7	91.5	91.7	91.1
	Ethylene Yield	65.8	46.7	35.4	28.8	22.5	19.4	18.1
	Diethyl Ether Yield	13.3	21.4	26.4	30.0	32.8	34.6	34.2

Note: Diethyl Ether Yield cannot surpass 50 mol%, as it is calculated based on moles of ethanol, as described in the experimental section of the main part of the paper.

References

1. S. Brunauer, P. H. Emmett and E. Teller, *Journal of the American Chemical Society*, 1938, **60**, 309-319.
2. E. P. Barrett, L. G. Joyner and P. P. Halenda, *Journal of the American Chemical Society*, 1951, **73**, 373-380.
3. M. E. Potter, A. E. Oakley, J. J. M. Le Brocq, L. N. Riley, M. Carravetta, S. M. King, C. M. Doherty, B. D. Vandegehuchte and R. Raja, *Journal of Materials Chemistry A*, 2023, **11**, 22822-22834.
